# Genomics of Signaling Crosstalk of Estrogen Receptor α in Breast Cancer Cells

**DOI:** 10.1371/journal.pone.0001859

**Published:** 2008-03-26

**Authors:** Peter Dudek, Didier Picard

**Affiliations:** Département de Biologie Cellulaire, Université de Genève, Genève, Switzerland; Institute of Genetics and Molecular and Cellular Biology, France

## Abstract

**Background:**

The estrogen receptor α (ERα) is a ligand-regulated transcription factor. However, a wide variety of other extracellular signals can activate ERα in the absence of estrogen. The impact of these alternate modes of activation on gene expression profiles has not been characterized.

**Methodology/Principal Findings:**

We show that estrogen, growth factors and cAMP elicit surprisingly distinct ERα-dependent transcriptional responses in human MCF7 breast cancer cells. In response to growth factors and cAMP, ERα primarily activates and represses genes, respectively. The combined treatments with the anti-estrogen tamoxifen and cAMP or growth factors regulate yet other sets of genes. In many cases, tamoxifen is perverted to an agonist, potentially mimicking what is happening in certain tamoxifen-resistant breast tumors and emphasizing the importance of the cellular signaling environment. Using a computational analysis, we predicted that a Hox protein might be involved in mediating such combinatorial effects, and then confirmed it experimentally. Although both tamoxifen and cAMP block the proliferation of MCF7 cells, their combined application stimulates it, and this can be blocked with a dominant-negative Hox mutant.

**Conclusions/Significance:**

The activating signal dictates both target gene selection and regulation by ERα, and this has consequences on global gene expression patterns that may be relevant to understanding the progression of ERα-dependent carcinomas.

## Introduction

In reproductive tissues, estrogens and peptide growth factors (GFs) are mitogenic and play important roles in the normal and aberrant development. The first indication that these signaling pathways communicate with each other was the observation that serum, insulin and IGF-I can stimulate ERα activity [1]. Indeed, ERα has been shown to be activated as a transcription factor by many signaling pathways even in the absence of its cognate ligand estrogen ([2]; and http://www.picard.ch/downloads/downloads.htm).

The biological cooperation between the EGF and IGF-I receptor pathways and ERα is the best studied to date. Antibodies against EGF inhibit estrogen-induced proliferation of uterine tissue [3] and the ER antagonist ICI164,384 reduces the response to EGF [4]. This relationship was confirmed genetically, since studies with ERα knockout mice showed a requirement for ERα for EGF-induced uterine growth [5], [6]. Moreover, the estrogen-dependent proliferation of the uterine stroma is defective in EGF receptor knockout mice [7]. In cultured cells, IGF-I is able to stimulate the transcriptional activity of ERα in the absence of hormone [8], [9]. The estrogen-independent activation of ERα by EGF requires the direct phosphorylation of ERα by MAPK [10] and this allows the recruitment of both positive and negative coregulators [11].

Elevated levels of cAMP also activate ERα in a ligand-independent fashion, but little is known about the mechanism of this response [12], [13]. Dopamine D1 receptor agonists like dopamine, which lead to increased levels of cAMP and activation of protein kinase A (PKA), have been shown to activate ERα in the absence of hormone [14]. PKA has been shown to modulate ERα function by phosphorylating the ERα residues S167, S236 and S305 [15]–[18]. However, there is no evidence that the PKA-elicited hormone-independent activation of ERα is a consequence of direct phosphorylation [19], and we have recently found that this activation is mediated by the phosphorylation-induced interaction with a transcriptional coactivator (P.D. et al., unpublished results).

Although the role of estrogen signaling for breast cancer has been extensively studied, it is not clear to what extent ligand-independent activation of ERα contributes to breast cancer progression to estrogen-independent or endocrine therapy-resistant forms. Overexpression or activation of the EGF receptor ErbB2 (also known as HER2 and Neu) or activation of MEKK1 has been shown to lead to resistance to the anti-estrogen tamoxifen in cell culture ([20]–[22], see also ref. [23]). Inhibition of ErbB2 or the p42/44 MAP kinases Erk1/2 restored the inhibitory effects of tamoxifen, underlining the importance of the MAPK pathway in regulating cellular growth in these systems [24]. Indeed, in clinical samples, Erk1/2 have been observed to be hyperactive and overexpressed in malignant breast tumors [25] and their activity correlates with a poor response to endocrine therapy and decreased survival of patients [26]. Furthermore, breast tumor-derived cells that exhibit elevated MAPK expression are hypersensitive to estradiol [27]. Interestingly, the ER coactivator SRC3 is amplified in breast and ovarian cancers [28], and is itself a target of the p42/44 MAPK pathway [29]. In a retrospective clinical study on tamoxifen treatment, co-overexpression of SRC3 with ErbB2 was correlated with the poorest outcomes in patients treated with tamoxifen [30].

The cAMP/PKA pathway may also contribute to endocrine resistance. Indeed, higher levels of cAMP-binding proteins were isolated from breast tumor samples that were resistant to endocrine therapy, compared with those that were not, suggesting the presence of a very active cAMP/PKA pathway [31]. A gene profiling study showed that the expression of one of the regulatory subunits of PKA, RIα, is significantly reduced in primary breast tumor samples and correlates with tamoxifen insensitivity [32]. This may be due to a hyperactive PKA phosphorylating ERα on S305 and locking it into a different conformation in the presence of tamoxifen [17], [18].

A considerable number of studies have been published that used DNA microarray technology to characterize the gene expression responses of breast cancer cells to estrogens and anti-estrogens (for example, refs. [33]–[38]). Except for one study that compared the GF responses of the uterus of ERα knock-out and wild-type mice [39], the impact of ligand-independent activation of ERα has not been investigated at the genomic level. We used microarray technology to determine whether different modes of activation of ERα lead to similar or different genomic responses and how those are affected by tamoxifen in human MCF7 breast cancer cells. We then performed a computational analysis on a set of target genes to predict the involvement of additional transcription factors and experimentally confirmed that some are responsible for the proliferative stimulus of the combined treatment of MCF7 cells with cAMP and tamoxifen.

## Results

### Genomic estrogen, cAMP and GF responses

**Figure 1:**
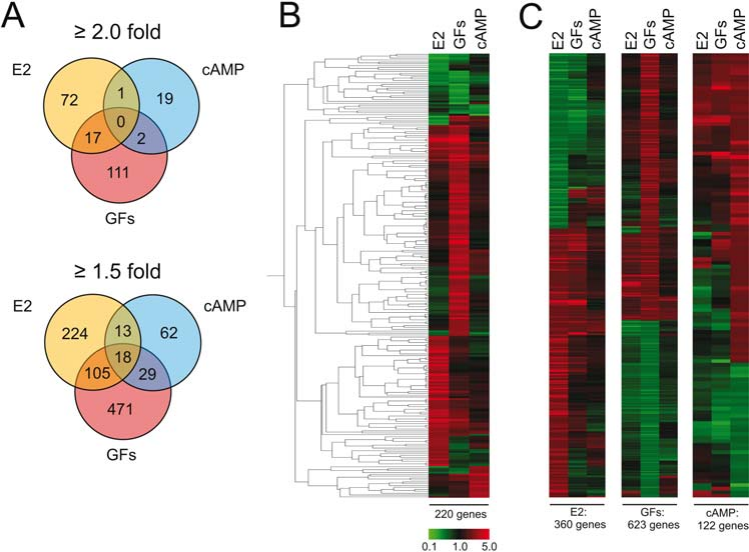
Analysis of gene expression profiles in response to different signals. (A) Venn diagrams of gene sets regulated by E2/GFs/cAMP. Top and bottom panels: genes regulated by at least 2-fold (total of 220 genes) and 1.5-fold (922 genes), respectively. (B) Hierarchical clustering analysis with Eisen tree of 2-fold gene set. (C) Clustering analysis using the gene sets defined separately by a 1.5-fold cut-off for each treatment. Green and red colour bars represent repression and induction, respectively, compared to control (untreated). Black bars indicate no change.

Recent studies concluded that the ERα-positive human breast carcinoma cell line MCF7 is an excellent model system for gene expression studies since the gene expression profiles are nearly identical to ERα-positive breast tumor xenografts and primary tumors [37]. Here we analyzed signaling crosstalk-specific profiles with MCF7 cells using a human cDNA array. To ensure that the expression profiles would be due to primary transcriptional responses, cells were induced for only 4 hours and in the presence of the translation inhibitor cycloheximide. Details on the experimental design and methodology are given in Supporting Information Text S1, and the treatments are listed and illustrated in Supporting Information Table S1 and Figure S1A, respectively. 220 unique transcripts of the 9,480 represented on the cDNA arrays responded to at least one of the three treatments by at least 2-fold (Figure 1A and Supporting Information Table S2). The genes regulated in the sample treated with 17β-estradiol (E2) accounted for 90 (40.9%) of all regulated genes, including many genes known to be regulated by E2 such as *PGR* (progesterone receptor gene), *BCL2*, *FOS* and *GREB1*. Other genes known to respond to estrogens like the Cyclin D1 gene (1.6-fold, p = 0.007) and *TFF2* (1.38 fold, p = 0.03), also responded to E2 in our experiment, albeit more weakly. The cAMP-regulated target genes accounted for 9.1% (20 of 220) of the total number of regulated genes. The known cAMP-responsive genes encoding p27/Kip1 (1.54 fold, p = 0.009) and Atf3 (1.65 fold, p = 0.012) also scored positive albeit below the 2-fold cut-off. The GF-treated sample showed the highest differential expression patterns compared with the control, accounting for 59.1% (130 of 220) of all genes regulated at least 2-fold, and the largest proportion of genes regulated at least 1.5 fold (623 of 922, or 67.6%). Classical EGF and IGF-I regulated genes such as *FOS* and *ERBB3* showed strong responses to the GF cocktail. Figure 1B represents a hierarchical clustering analysis and Eisen tree of the responses of the 220 genes that exhibited expression differences of at least 2-fold in the E2, cAMP and EGF/IGF-I treated samples. Figure 1C represents three clustering analyses of the genes affected at least 1.5-fold by each treatment: E2 (360 genes), EGF/IGF-I (623 genes) and cAMP (122 genes), with a total of 922 unique transcripts.

### Validation of some microarray results by quantitative real-time RT-PCR

**Figure 2:**
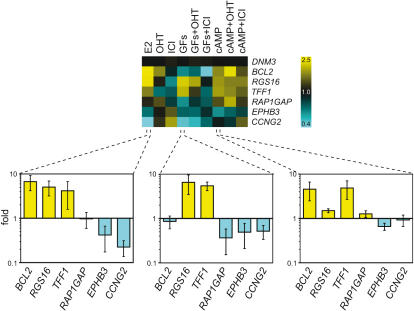
Verification of microarray data by Q-PCR. Top panel: dendrogram of fold expression ratios from the microarray data of the genes *DNM3, BCL3, RGS16, TFF1 (pS2), RAP1GAP, EPHB3* and *CCNG2*. Yellow and cyan indicate induction and repression, respectively. Bottom panel: Q-PCR results for three conditions (E2, GFs and cAMP). Log-scale graph of the fold expression values calculated with the ΔΔCt method using *DNM3* as internal control. Values shown are the means of triplicate samples ± standard deviation.

In order to verify the gene expression patterns observed in the microarray analysis, several genes with diverse patterns were selected and confirmed by Q-PCR. We chose two genes known to be regulated by E2 (*BCL2* and *TFF1/pS2*), and several others with interesting expression patterns and potential relevance to cell proliferation and cancer, including *RGS16* (Regulator of G protein signaling 16), *RAP1GAP* (Rap1 GTPase activating protein 1), *CCNG2* (Cyclin G2) and *EPHB3* (*Ephrin type-B receptor 3 precursor)*. As an internal control, *DMN3* (Dynamin 3) was selected because of its abundant expression in all samples and the absence of detectable differences in expression under all treatment conditions. The results from three conditions (E2, GFs, and cAMP) are shown in Figure 2 along with a dendrogram representing the microarray data for comparison. Although in some cases the quantitative expression values from the PCR results differed considerably from the microarray data, the results are qualitatively identical. Thus, the Q-PCR results validated the gene expression patterns of the genes tested.

### ERα-dependent gene regulation via crosstalk with cAMP and GFs

Having established that a subset of genes is regulated independently by several treatments, we determined for which of the genes the regulation by cAMP or EGF/IGF-I is ERα-dependent. ERα-signaling can be blocked with the pure anti-estrogen ICI 182,780 (ICI), and therefore combining it with cAMP or EGF/IGF-I should specifically affect ERα-dependent genes, including those regulated by cAMP or GFs. We quantitatively defined an ERα-dependent gene as one for which the difference of expression with the addition of ICI was at least 20%. E2+ICI and E2+hydroxytamoxifen (OHT) treatment controls were deliberately not included since these kinds of data already abound in the literature, and since the focus of our study was on signaling crosstalk and not on (re-)identifying genes regulated by E2- or OHT-activated ERα. It must be emphasized that ICI does not act as an antagonist for the novel membrane-associated estrogen receptor GPR30 [40]–[42] allowing us to use this pharmacological criterion to define ERα-dependence.

**Figure 3:**
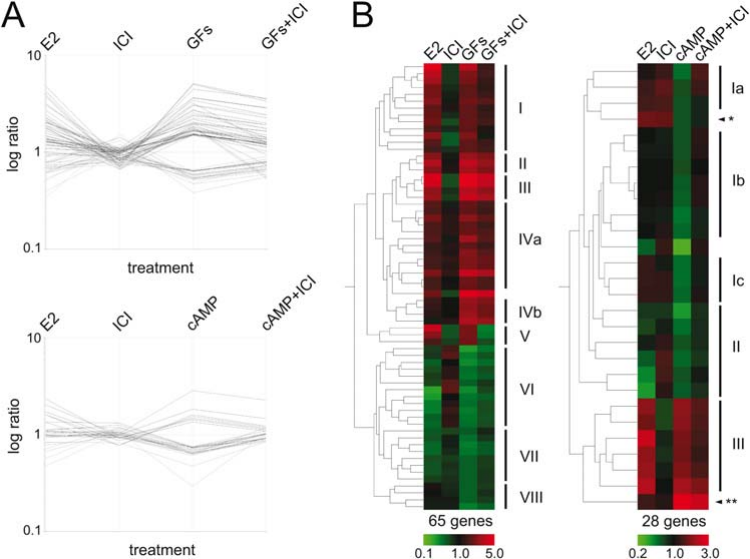
Ligand-independent ERα-mediated gene expression profiles. (A) Log expression ratios of the ICI-affected GF-regulated (65 genes) and cAMP regulated (28 genes) gene sets at the top and bottom, respectively. (B) Hierarchical cluster analysis with Eisen tree of gene sets shown in panel A. Interesting clusters are numbered on the right. Asterisks denote genes with unique response that are discussed in the text. Colour code as in Figure 1.

In total, 65 of 623 (10.4%) GF-regulated genes were affected by ICI treatment (Figure 3A top and 3B left; Supporting Information Table S3). The genes appear to organize into 8 unique clusters, labelled I–VIII. Cluster I represents genes that are independently induced by E2 and GFs and repressed by ICI alone. Repression by the sole treatment with ICI suggests that these are genes with a strong ERα basal activity. Cluster II differs from I by the fact there is no difference in the ICI-only sample and hence no basal ERα activity. The behavior of the genes in cluster III is similar to those of cluster I, differing only in the absolute expression values. Cluster IV represents genes that, by and large, are not significantly induced by E2 (if at all), unaffected in the ICI-only sample but induced by GFs in an ERα-dependent fashion. Cluster V corresponds to GF-induced genes most heavily affected by ICI treatment, i.e. the genes with the strongest dependence on ERα for induction. In contrast to clusters I–V, clusters VI–VIII collectively correspond to genes that are repressed by GF treatment and derepressed by ICI. Cluster VI includes genes with a significant difference in the ICI-only sample, whereas cluster VII does not. However, both VI and VII contain genes that are repressed by E2. Cluster VIII resembles IV in that neither E2 nor ICI-alone affects expression. Clusters IV and VIII are examples of genes that are regulated by ERα exclusively upon activation by GFs and not by E2.

With the original more stringent 2-fold cut-off, the set of cAMP-regulated genes was relatively small. We therefore lowered the threshold to 1.3-fold to obtain a larger number of ERα-dependent genes (Figure 3A bottom and 3B right; Supporting Information Table S3). Of the 28 genes, 26 could be classified into three unique clusters. The two remaining genes, *LMCD1* and *RAFTLIN*, exhibited their own unique behaviors. All three clusters exhibited no significant changes in expression in the ICI-only sample. Cluster I is the largest of the classes, accounting for half of the genes in this set, and representing genes that are repressed by cAMP in an ERα-dependent way but not affected by E2. Much like for clusters IV and VIII from the GF gene set, ERα appears to be required but not sufficient to regulate these genes, at least in the presence of E2. Cluster II represents genes that are repressed by both E2 and cAMP. In contrast, cluster III corresponds to genes that are induced by both E2 and cAMP. The gene *RAFTLIN* (double asterisk) is the only example of a gene for which E2 had no effect and yet cAMP induced it in a partially ERα-dependent fashion. The gene *LMCD1* (single asterisk) is the only one in either ERα-dependent gene set to exhibit opposite responses to hormone and to signaling crosstalk.

### Effects of cAMP and GFs on the tamoxifen response

Unlike ICI, OHT is not a pure anti-estrogen and can even activate ERα in a context-dependent manner. Thus, in addition to studying the genomic effects of ligand-independent activation of ERα, we wished to understand how signaling crosstalk can influence the intrinsic agonistic or antagonistic activities of OHT at the genomic level. We defined an OHT-coregulated gene (as opposed to a gene that is affected by OHT alone) as one where OHT-cotreatment resulted in an expression difference of at least 1.3-fold compared to cAMP/GFs alone. However, if a gene exhibited the same behavior in the OHT-alone sample as in the cotreatment sample, then it was filtered out and not further considered in the analysis. In addition, in order not to confuse, for instance, inverse agonism with antagonism, the cotreatment sample genes were filtered out if they behaved in the opposite fashion in the presence of E2. This was the case, for example, for genes that are repressed by E2 but induced by the addition of both OHT and cAMP or GF. Although we cannot formally exclude the possibility that some OHT (or E2) signaling effects in MCF7 cells were mediated by ERα-independent pathways such as those regulated by GPR30, the focus on genes differentially affected by the combined treatments of OHT with cAMP or GFs (based on the “filtering” mentioned above) would tend to exclude a major contribution of those. Moreover, preliminary results from profiling GPR30-mediated gene expression changes in response to OHT indicate an entirely different set of target genes than the ones studied here (Deo Prakash Pandey, Marcello Maggiolini, and DP, unpublished results).

**Figure 4:**
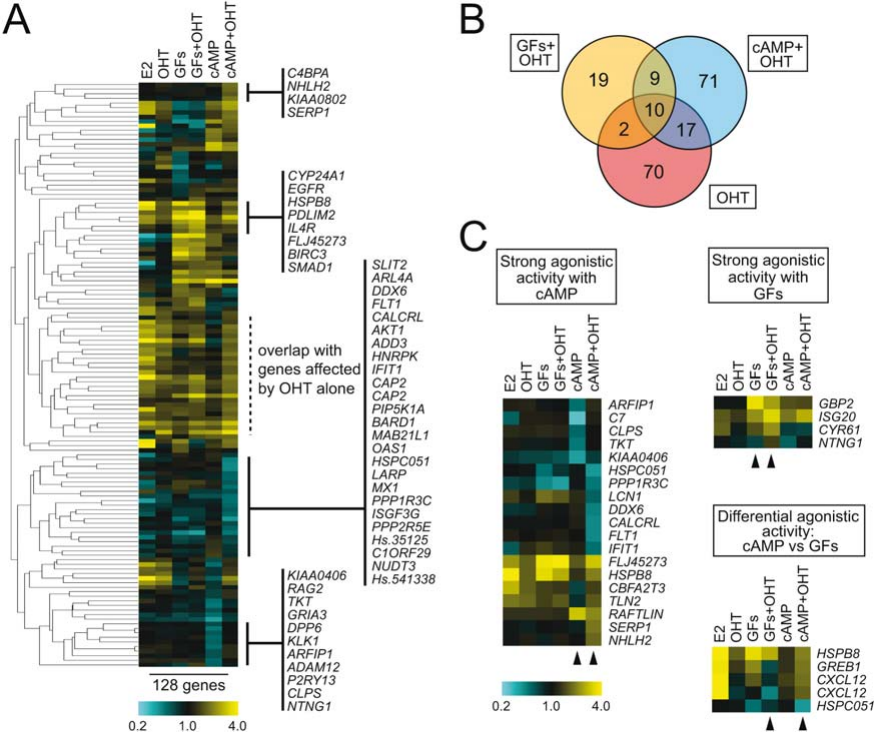
Interplay between OHT and signaling crosstalk. (A) Hierarchical cluster analysis with Eisen tree of genes affected by OHT in GF- and cAMP-treated samples. Gene set includes 20 genes that also respond in the same manner to OHT alone (marked with dotted lines). Some sets of genes with interesting behaviors are marked with solid lines and listed. (B) Venn diagrams illustrating overlap between gene sets obtained with OHT alone or in combination with cAMP or GFs. (C) Selected patterns of genes most strongly affected by the agonistic activity of OHT. Arrows mark rows of most relevant pairwise comparisons.

OHT was found to coregulate 80 genes in concert with cAMP compared to only 28 genes with GFs (Supporting Information Table S4, and Figure 4B). This was an unexpected finding considering the proportion of genes that cAMP and GFs regulate when applied separately, and might suggest that cAMP is significantly more effective than GFs at modulating OHT activity. A cluster analysis of these genes is shown in Figure 4A. To highlight the importance of filtering out those genes that display the same behavior with OHT alone, this analysis, as an exception, still includes them. There is remarkably little overlap between the responses to these different treatments (Figure 4B), illustrating a crosstalk-specific agonistic activity for OHT that differs substantially from its intrinsic agonistic activity. It should be noted that the term agonist is used here to denote a ligand, OHT, that turns on ERα activity above its basal activity, irrespective of whether it then acts as a repressor or an activator of transcription. Genes where OHT showed the strongest agonistic activity are illustrated in Figure 4C, and are shown separately for both crosstalk signals. Interestingly, there was a small group of genes in the original set of 108 OHT-coregulated genes (80 coregulated with cAMP plus 28 coregulated with GFs) that exhibited the intriguing behavior of opposing responses when comparing the two crosstalk signals (Figure 4C). Whereas OHT induces the expression of *HSPB8*, *GREB1* and *CXCL12* in the cAMP-treated sample, it represses it in the GF-treated sample. Similarly, OHT derepresses the GF-induced repression of *HSPC051*, whereas it represses the same gene in the cAMP-treated sample. Since these opposing behaviors occur with the same genes, it implies that there are two independent molecular mechanisms of OHT-mediated signaling crosstalk.

### Computational analysis of promoters involved in ERα-dependent, cAMP-regulated gene regulation

**Figure 5:**
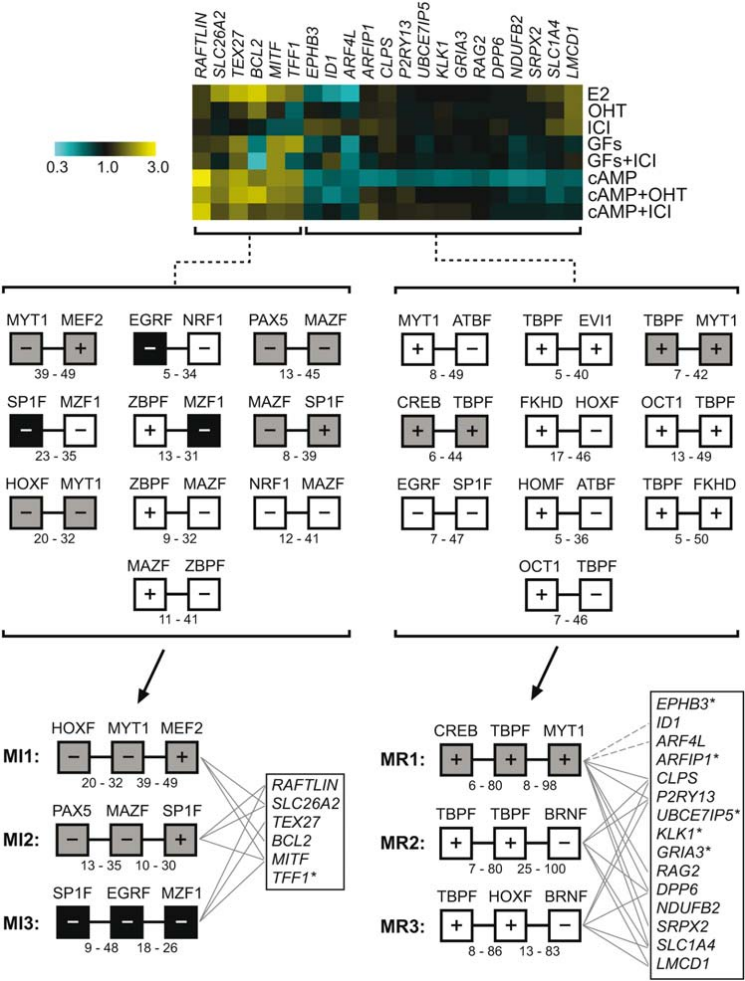
Computational modelling of transcription factor binding site modules in ERα-dependent/cAMP-regulated promoters. Top panel: microarray data of those genes from this set for which promoter sequences (−1000 bp to +100 bp) were available (21 out of 28). Middle panel: list of ten highest scoring 2-element transcription factor binding site (2xTFBS) modules. Bottom panel: list of three optimized modules containing 3 transcription factor binding-sites (3xTFBS) each; hashed lines, partial module coverage; grey boxes, conserved 2xTFBS module transmitted to a 3xTFBS module; black box, conserved 1xTFBS transmitted to a 3xTFBS module; asterisks denote genes with no detectable 3xTFBS modules. + and − indicate strand orientation, and digits indicate distances in base pairs between transcription factor elements.

The ligand-independent activation of ERα by cAMP leads to the regulation of a distinct set of genes (Figure 3B, right panel) whose responses to various other treatments are highlighted in Figure 5 (top panel). We hypothesized that the regulation of the ERα-dependent/cAMP-regulated genes might be explained by a simple combinatorial code of transcription factor co-recruitment that could be detected by analyzing the promoter/enhancer regions of these genes. To facilitate the computational promoter analysis, we employed ModelInspector [43], a component of the GenomatixSuite software. Of the 28 unique transcripts that were cAMP-regulated and affected by ICI treatment (ERα-dependent) in our microarray data set, 21 were identifiable genes whose promoter sequences could be downloaded and analyzed. These sequences were scanned with ModelInspector for matches to a predefined library of functional promoter modules. A “module” is defined by a combination of two to three specific transcription factor binding sites, their order, strand orientation and distance range. The best scoring 2-element and 3-element transcription factor binding sites (modules) are illustrated in Figure 5. Most importantly, no single 2-element or 3-element module was found to occur in all promoters from either the upregulated or repressed group of genes. The top three 3-element modules in the cAMP-induced group (MI1, MI2 and MI3) occurred in 3 out of 6 of promoters, but 5 out of 6 promoters were covered by a combination of MI1 and MI3. Only the *MITF* promoter contained all three modules. In the cAMP-repressed group, fewer common 3-element modules were uncovered than in the cAMP-induced group (3 versus 8), despite there being significantly more promoters to scan; this may reflect a more robust functional conservation of the respective promoters. The MR2 and MR3 modules were found in 5 out of 15 promoters each, whereas the MR1 module occurs in 8 promoters, though two of those promoters, belonging to the *ID1* and *ARF4L* genes, contain a poorly conserved MYT1 site (marked with dotted lines in Figure 5). There is significant overlap among the 3 modules, and yet, one third of the promoters in this group had no detectable 3-element module. Results from a genome-wide search of these modules in other promoters as well as a gene ontology analysis are summarized in Supporting Information Tables S5-B and S5-C.

### Requirement for a homeodomain-containing protein for cAMP/OHT-dependent proliferation of MCF7 cells

**Figure 6:**
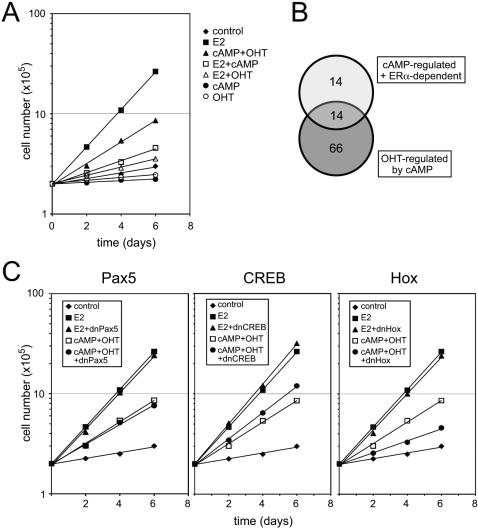
A Hox protein mediates cAMP-induced agonistic switch of OHT for MCF7 proliferation. (A) MCF7 proliferation assay. Equal numbers of cells (2×10^5^) were seeded onto plates and then treated over a period of 6 days with vehicle control (0.1% ethanol), 10 nM E2, 1 mM 8-Br-cAMP (cAMP), 1 µM OHT or combinations thereof. (B) Venn diagram showing overlap between the cAMP-regulated/ERα-dependent genes and genes OHT-regulated by cAMP (corrected for E2 and OHT behavior as described in the text). The overlapping 14 genes include the 11 genes used in the promoter analysis of Figure 5 (*ARF4L, ARFIP1, CLPS, DPP6, GRIA3, KLK1, NDUFB2, P2RY13, RAFTLIN, RAG2, UBCE7IP5*) and the unannotated transcripts/genes KIAA0406, KIAA0556 and Hs.541338 (see Supporting Information Tables S3 and S4). (C) Effect of dominant-negative transcription factor mutants on proliferation of MCF7 cells. Cells were transfected with expression vectors for dnPax5, dnCREB or dnHox, or empty expression vector (control). Data in panels A and C are representative of two independent experiments.

Much like anti-estrogens such as OHT, cAMP is known to inhibit E2-dependent proliferation of MCF7 cells by inhibiting the G1/S transition [44]–[46] (see also Figure 6A). Remarkably, the antiproliferative activity of cAMP is partially rescued with OHT (Figure 6A), which may be due to cAMP-induced OHT-agonism. Intriguingly, about half of the genes (11 out of 21) used in the promoter analysis of ERα target genes regulated by cAMP are also genes whose expression was subject to cAMP-induced OHT-agonism (Figure 6B). Therefore, we hypothesized that one or more of the transcription factor binding site modules found earlier might be involved, in conjunction with ERα, in the transcriptional regulation of a gene (or genes) important for proliferation induced by the combined treatment with cAMP and OHT.

To test this, we overexpressed dominant-negative (dn) forms of Hox, Pax5, and CREB in MCF7 cells (representing the modules MI1, MI2 and MR1, respectively), and measured the proliferative responses to OHT and cAMP (Figure 6C). Neither overexpression of dnPax5 nor dnCREB blocked cAMP/OHT-induced proliferation. dnCREB even slightly promoted proliferation, possibly because CREB is the target and mediator of the anti-proliferative cAMP pathway. In contrast, overexpression of dnHox significantly inhibited cAMP/OHT-induced proliferation whereas it had no effect on E2-induced proliferation. dnHox was derived from HoxD13, but since it essentially only contains the homeodomain, we expect it to function as a dominant-negative mutant for many homeodomain-containing transcription factors. Thus, these results suggest that an as yet unidentified homeodomain-containing transcription factor is a mediator of the cAMP/OHT-induced proliferation of MCF7 cells.

## Discussion

The key finding of our comparative microarray analysis is that cAMP, growth factors and estrogen elicit vastly different transcriptional responses, both at the level of the whole genome and, perhaps more remarkably, of ERα-dependent genes. Thus, depending on how ERα is activated, it regulates largely different sets of genes. Another unexpected finding is that ERα primarily represses genes in response to cAMP despite the fact that this particular response was originally characterized with a reporter gene that monitors activation. Although a large number of microarray studies that focused on ERα and/or breast cancer cells had been reported (for example, refs. [33]–[39]), these aspects had been missed. Our analysis uniquely concentrated on signaling crosstalk with ERα, notably its ligand-independent activation, and on the agonistic effects of OHT in the presence of additional extracellular signals, which at least in part are also mediated by ERα.

### ERα-dependent gene expression and signaling crosstalk

Among the ERα-dependent groups of genes with varied behavior, clusters IV and VIII from the GF sample and cluster I from the cAMP sample are extremely interesting. They represent genes whose regulation by GFs or cAMP requires ERα, but for which the cognate ligand E2 has no effect. This is strong evidence that cAMP and GFs are activating ERα in a ligand-independent manner. Moreover, it argues that ligand-independent activation can lead to a specific gene expression response, which differs markedly from the hormone-dependent response. The ligand-independent response also varies depending on the source of the signal, since there is virtually no overlap between the ERα-dependent genes regulated by cAMP and those regulated by GFs. Indeed, to our surprise, among the 96 unique transcripts that were ERα-dependent in the crosstalk samples, only one gene, *TFF1(pS2)*, appeared in both the cAMP and GF group (see also top panel in Figure 5). This supports the idea that cAMP and GF-signaling lead to different ERα-dependent outcomes, and suggests that two distinct ligand-independent mechanisms of activation are at play.

Moreover, there are interesting differences between the two signaling crosstalk responses. For instance, there is not a single example of an ICI-affected gene that is repressed by E2 and induced by GFs (or vice versa). When E2/GF-regulation requires ERα, it appears to be always unidirectional. This does not seem to be the case with E2/cAMP regulation. *LMCD1*, for instance, is weakly induced with E2 alone, but repressed by cAMP, and yet, both responses require ERα. Another difference between the two ligand-independent responses is the relative distribution of induction and repression of gene expression. Whereas GFs activate ERα primarily to induce gene expression, it appears that cAMP activates ERα to repress the expression of genes. Unfortunately, still relatively little is known about how E2-liganded ERα represses genes [47]-[49]. Hence, it is difficult to begin to speculate about the molecular mechanisms.

A first indication about the underlying molecular mechanisms that direct signal-specific responses comes from a recent study by Surmacz and colleagues [50]. Using chromatin immunoprecipitation experiments, they found that different coactivator complexes are assembled on two selected ERα target genes in response to E2 or IGF-I. The nature of these complexes was both signal-dependent and influenced by whether ERα was recruited to the target gene directly through an estrogen response element or indirectly through another transcription factor (AP1). Taken together with our own genomic analysis, this argues that different signals activate ERα to do different things, either on different genes or even on the same gene. At the molecular level, this means that ERα assembles different coactivator (or corepressor) complexes as a function of target gene and activating signal. It is also conceivable that different signals already direct ERα to different target genes. Future experiments will address these mechanistic aspects as well as the physiological consequences of these signal-specific responses.

Our findings seem to contradict those found by the only other microarray study that specifically looked at ERα/GF signaling crosstalk, and which concluded that the GF response is not altered by the deletion of the ERα gene in the mouse [39]. While it was difficult for these authors to reconcile these results with their previous demonstration that the growth response of the uterus to GFs is mediated by ERα [5], [6], it has not been clarified whether estrogen and GFs both crosstalk *and* act in parallel in this system and to what extent these are responses specific to the uterus.

### Agonistic activity of tamoxifen and implications for endocrine resistance

Our findings may also have implications for endocrine therapy. A large proportion of ER+ breast cancers eventually become resistant to anti-hormone treatment [51]. There is also a large body of evidence that directly implicates the aberrant behavior of growth factor receptor pathways, in particular the ErbB2 and MAPK pathways, in the acquisition and maintenance of tamoxifen resistance (discussed in ref. [2]). Furthermore, very few studies have been done on the role of cAMP signaling for anti-hormone resistance, despite first evidence as early as 1990 [31]. Although cAMP/PKA signaling has been shown to pervert tamoxifen to an agonist for ERα target gene activation [17], [52], the molecular mechanisms remain unclear. Nevertheless, our analysis further illustrates that studying the effects of OHT on gene transcription in isolation may not yield an accurate picture of its biological activity *in vivo* and highlights the importance of considering the cellular signaling environment as a whole when interpreting such results.

The computational analysis of signal-specific ERα target gene sets did not reveal a single common transcription factor framework that could reliably explain or predict the observed genetic behavior. This may indicate a multi-layered complexity that would require further experimentation to separate. Despite this, several highly specific promoter modules containing three transcription factor binding sites separated by a specific distance with a specific strand orientation were found in ERα target genes differentially regulated by cAMP. Three modules were revealed that contain highly conserved Hox, Pax5 and CREB transcription factor binding sites. We addressed the physiological significance of this *in silico* association with a MCF7 proliferation assay. It was known that elevation of cAMP suppresses E2-dependent proliferation of MCF7 cells [44]–[46], but not of tamoxifen-resistant MCF7-LCC2 cells [46]. We observed that cAMP in combination with OHT promotes the proliferation of MCF7 cells, albeit at less than 50% the effectiveness of estrogen. This is the first time that the potentially adverse consequences of cAMP signaling crosstalk for endocrine therapy are recapitulated by augmenting cAMP levels in tissue culture, and not just for the activation of one or two ERα target genes. It confirms a previous report that showed a similar growth stimulation by knocking down expression of a regulatory subunit of PKA [17]. Furthermore, overexpression of a dominant-negative Hox protein, harboring only the homeodomain, was sufficient to block this cAMP/OHT-induced proliferation. This result implicates a homedomain-containing transcription factor as a transcriptional facilitator of OHT-agonism. By “facilitator” we mean that the Hox protein(s) could be required to allow the regulation of genes required for proliferation by ERα in the presence of cAMP and OHT. This does not need to involve any kind of direct interaction between a Hox protein and ERα, but instead the Hox protein might merely prepare a promoter or enhancer for regulation by ERα. Although the computational analysis strongly suggests some kind of functional interaction, it is formally conceivable that a Hox protein acts through an unrelated (ERα-independent) gene in the presence of cAMP to synergize with OHT. Such mechanistic questions will be addressed most efficiently once the Hox protein(s) will have been identified. It will also be interesting to evaluate whether a direct phosphorylation of this Hox protein(s) by PKA, as is known for the paradigmatic PKA-regulated transcription factor CREB [53], contributes an additional layer of regulatory complexity.

Hox proteins are a large family of transcription factors. The most common element among all factors is the highly conserved homeodomain, which is a 61 amino acid helix-turn-helix DNA binding motif. In addition, over 100 other human proteins contain the homeodomain as well. *HOX* genes are important in embryogenesis and organogenesis because they regulate cell proliferation and differentiation. Since normal development and cancer involve a balance between proliferation and differentiation, it is not surprising that *HOX* genes have been linked to many different tumors, including breast cancer [54]. Expression studies of the entire *HOX* gene cluster in breast cancers revealed elevated expression for several members, including *HOXA4*, *B3*, *B13*, *C13*, *D3* and *D13*. Moreover, *HOXB13* overexpression can be used as a component of a two-gene signature predicting poor outcome in tamoxifen-treated patients, and its ectopic expression promotes motility and invasion *in vitro* [55]. It remains to be determined which Hox protein is the one that is required for the proliferation of breast cancer cells exposed to both tamoxifen and signals elevating cAMP.

Our analysis provides a genomic glimpse into how cAMP and GFs pervert tamoxifen into an agonist, and sets the stage for identifying and characterizing genes involved in causing resistance to endocrine therapy. It will also have to be established what the source of signals may be that leads to elevated levels of cAMP and GFs. Apart from autocrine or paracrine signals from the epithelial cells themselves, a stromal origin should be considered. The recent discovery that mesenchymal cells promote the metastasis of breast cancer cells is a telling illustration [56]. At least for a subset of ERα-dependent carcinomas, our genomic analyses emphasize the necessity to consider the impact of additional extracellular signals on the effectiveness of tamoxifen as an antagonist. Our results might ultimately facilitate the development of personalized forms of endocrine therapy.

## Materials and Methods

### Cell culture

An estrogen-independent (but not ERα-independent) MCF7 variant MCF7-SH [57] was used for microarray and Q-PCR experiments. Because this MCF7 variant expresses higher levels of ERα, transcriptional responses could be expected to be more robust. These cells were grown in phenol red-free Dulbecco's modified Eagle's medium (DMEM) supplemented with 10% (v/v) charcoal-treated fetal calf serum (FCS). For proliferation assays, estrogen-*dependent* MCF7 cells (a gift from F. Auricchio, Naples) had to be used. They were maintained in medium supplemented with E2. Note that both of these types of MCF7 cells express ERα but not ERβ (data not shown).

### Microarray analysis

The human 10K E cDNA arrays, generated by the DNA Array Facility of the University of Lausanne, Switzerland (DAFL), are based on the Incyte cDNA collection (for details, see GEO accession number GPL2746). Treatments for expression analyses were for 4 hours with 100 nM 17β-estradiol (E2), hydroxytamoxifen (OHT) or ICI182,780 (ICI), 1 mM 8-Br-cAMP or a combination of 50 µg/ml EGF and 50 µg/ml IGF-I, in the presence of cycloheximide. The detailed procedures for treating the cells, isolation of RNA, and production of Cy5- and Cy3-labelled cDNA probes of treatment and control samples, respectively, are described in Supporting Information Text S1 and Figures S1 and S2. Cluster analysis was done using the software Genespring (Agilent), and promoter analysis was done with the GenomatixSuite software (www.genomatix.de), notably ModelInspector and the proprietary Promoter Module Library. The microarray data were deposited with GEO (accession number GSE10466).

### Quantitative real-time PCR analysis

Real-time RT-PCR was done as described in Supporting Information Text S1 with the same total RNA sample replicates used in the microarray experiments. Primers are listed in Supporting Information Table S6.

### Dominant negative constructs, MCF7 transfection and proliferation assay

The expression vector pKW-prd-en for dominant negative Pax5 was a gift from M. Busslinger [58]. The plasmid for A-CREB was a gift from C. Vinson [59]. A construct with exon 2 of HoxD13 in Bluescript (a gift from D. Duboule) served as template for PCR amplification of the homeodomain coding sequences with the primers 5′-GGGAATTCTGGATGTGGCTTTAAA-3′ and 5′-GGAATTCTCAGGAGACAGTGTCTTTG-3′. This PCR product was cloned into *Eco*RI-*Bam*HI-digested plasmid CKF [60]. Estrogen-dependent MCF7 cells were seeded into 6-well plates at 2×10^5^ cells per well in phenol red-free DMEM supplemented with 5% (v/v) charcoal-treated FCS and transfected using TransIT LT1 (Mirus) (day 0). Note that transfection efficiencies routinely reached 70–90%. 24 hours post-transfection (day 1), cultures were supplemented with hormones as required. Cells were counted on days 2, 4, and 6.

## Supporting Information

Text S1 (0.05 MB DOC)

Figure S1 (0.19 MB PDF)

Figure S2 (0.11 MB PDF)

Table S1 (0.03 MB PDF)

Table S2 (0.11 MB PDF)

Table S3 (0.06 MB PDF)

Table S4 (0.08 MB PDF)

Table S5 (0.06 MB PDF)

Table S6 (0.03 MB PDF)
