# Supplementary material for: Genomics of Signaling Crosstalk of Estrogen Receptor α in Breast Cancer Cells
Source: PLoS One. 2008 Mar 26;3(3):e1859. doi: 10.1371/journal.pone.0001859 (PMC2268000; doi:10.1371/journal.pone.0001859)
Supplement: Text S1 — (0.05 MB DOC) [file pone.0001859.s001.doc]

Supporting Information

Dudek and Picard

Further experimental details

**Experimental design**

Since we were interested in mapping the ERα-dependent transcriptional network regulated by GFs or cAMP, as well as their modifying contributions to the gene expression profile with tamoxifen, cells were subjected to a panel of treatment conditions (Supporting Information Table S1). To maximize the effects of the GF treatments as well as to minimize the pleiotropic effects of the culture serum, cultures were starved of serum for 24 hours before treatment. To ensure that the expression profiles obtained would be due to primary transcriptional responses, the cultures were co-treated with the translation inhibitor cycloheximide. The cytotoxicity of the cycloheximide treatment itself was assessed with a cell viability assay and suggested that the optimal treatment time before RNA isolation would be less than 5 hours (Supporting Information Figure S1). Thus, to maximize the effect of the experimental treatments on mRNA levels, and to avoid the toxic effects of a long-term exposure to the translation inhibitor, we chose a 4 hour treatment period for all samples. The experimental time-course is shown in Supporting Information Figure S1A, and a schematic diagram of the experimental design is given in Supporting Information Figure S2. In total, 5 independent experiments were performed, out of which three that exhibited the highest RNA quality were chosen as replicates (the remaining two discarded), totalling 30 RNA isolates (three for each condition) that were used for mRNA amplification, cDNA synthesis and labeling. The three control replicates were independently labelled with Cy3, and all other samples with Cy5. Each Cy5-labelled sample was then mixed with an equimolar amount of Cy3-labelled control, producing a dual-dye cDNA mixture for hybridization and 2-channel scanning. To maximize statistical robustness, each replicate was hybridized to a separate chip, totalling 27 cDNA slides representing 9 unique conditions.

**Cell culture, treatments and RNA isolation**

MCF7-SH cells were grown in phenol red-free Dulbecco's modified Eagle's medium (DMEM) supplemented with 10% (v/v) charcoal-treated fetal calf serum (FCS), 1 mM sodium pyruvate, 2 mM L-glutamine, 100 U/ml penicillin, 100 μg/ml streptomycin. 24 hours prior to treatment, cells were starved of serum. 30 min prior to induction, 50 μg/ml cycloheximide was added to all cultures. Cells were then washed 3x with phosphate-buffered saline prior to RNA extraction. Total RNA was extracted using Trizol (Invitrogen). This experiment was repeated 5 times. RNA quality was assessed with a BioAnalyzer (Bio-Rad). All RNA samples were stored at -80°C until required for further processing.

**Microarray analyses**

50 μg of RNA from each sample was used for each probe. mRNA was first amplified using the MessageAmp™ II aRNA amplification kit (Ambion). Labelled cDNA was subsequently prepared with the RT Superscript kit (Invitrogen), using 2 μg oligo-dT primers (Amersham Biosciences) and the following nucleotide concentrations: 0.5 mM dATP, 0.5 mM dGTP, 0.5 mM dTTP, 0.2 mM dCTP supplemented with 0.1 mM Cy5-dCTP (Amersham Biosciences) except for the control sample, which was supplemented with 0.1 mM Cy3-dCTP (Amersham Biosciences). After reverse transcription (RT), RNA was hydrolyzed with 25 mM EDTA and 1 M NaOH for 10 min at 65°C. The alkaline samples were then neutralized and buffered with 1 M HCl and 1 M Tris-HCl pH 6.8. The labelled probes were purified using a Qiagen MiniElute kit (Qiagen). To create the final probe mixture for hybridization, each Cy5-labelled treatment sample probe was mixed with an equimolar amount of the Cy3-labelled control sample probe and then concentrated to a volume of 19.4 μl using a Microcon YM-30 kit (Millipore) and subsequently mixed with a SSC buffer containing 0.4% SDS to a final volume of 24 μl. All hybridization reactions were carried out in an ozone-free environment. cDNA slides were placed into hybridization chambers (TeleChem International), and 10 μl of 3x SSC buffer was added to each groove in the chamber for humidifying. The mixed probes were heated for 1 min at 95°C, then spun in a microcentrifuge at maximum speed for 1 min. The entire volume of the probe was pipetted onto the slide and covered with a cover slip. The chamber was then tightly sealed and immersed for 18 hours in a 64°C bath. The slides were removed from the chambers and washed for 2x 5 min in 2x SSC + 0.1% SDS buffer, followed by 2x 1 min in 0.2x SSC buffer, followed by 1 min in 0.1x SSC buffer, followed by 5 min in 0.1x SSC + 0.1% Triton X-100. Slides were subsequently dried by centrifugation at 800 rpm for 2 min, and immediately scanned using an Agilent G2565B DNA Microarray Scanner. Quality control analysis and normalization was carried out by the DAFL. The cDNA array (GEO accession number GPL2746) consisted of ~10,000 spots, of which 9,480 were unique human transcripts and the remaining spots representing various quality controls from yeast and Arabidopsis. Responsive genes were determined by statistical analysis of the log expression ratios of treated samples versus the untreated control using a paired sample *t*-test with a p-value cutoff of 0.05.

**Q-PCR**

Reverse transcription was carried out using the Superscript III RT kit (Invitrogen). Real-time PCR was performed on an iCycler iQ5 with the iQ™ SYBR® Green Supermix reagent (Bio-Rad). Reactions were prepared in 96-well plates as follows: 12.5 μl SYBR green supermix, 500 nM each of forward and reverse primers, 5 μl of cDNA from the RT reaction, in a total volume of 25 μl per well. Primers were designed to ensure an amplicon size range of 100-130 bp, and a Tm of 59.5°C. Primer sequences are shown in Supporting Information Table S6. Three independent Q-PCR reactions were performed on the original total RNA isolates from the three experimental replicates chosen for the microarray analysis.

**Computational analyses of promoter sequences**

For the promoter analysis with the GenomatixSuite (www.genomatix.de), promoters were taken from the human Genomatix Promoter Database (GPD). Sequences 1000 base pairs (bp) upstream of the first transcription start site (TSS), and 100 bp downstream of the last TSS (if more than one existed) were extracted and separated into two distinct functional groups: cAMP-induced (6 promoters) and cAMP-repressed (15 promoters). Each group was then scanned for orientation and distance correlated transcription factor binding sites (termed "modules") (Supporting Information Table S5A).
