# Supplementary material for: Genomics of Signaling Crosstalk of Estrogen Receptor α in Breast Cancer Cells
Source: PLoS One. 2008 Mar 26;3(3):e1859. doi: 10.1371/journal.pone.0001859 (PMC2268000; doi:10.1371/journal.pone.0001859)
Supplement: Figure S1 — (0.19 MB PDF) [file pone.0001859.s002.pdf]

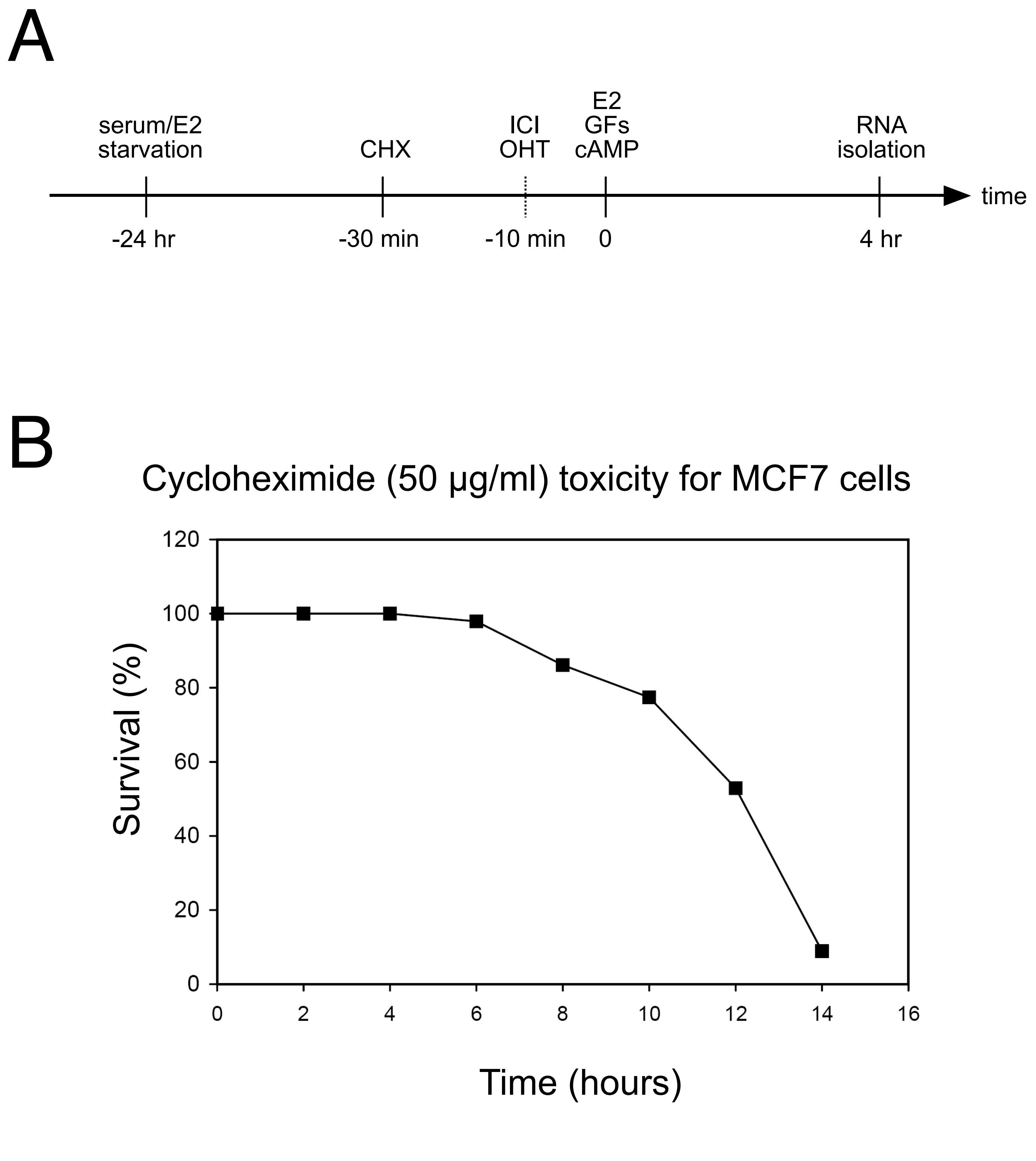

Supplementary Figure S1:

A, Scheme of experimental design. Experimental time course with details of cell culture treatments. CHX, cycloheximide. *B*, Cycloheximide toxicity assay with MCF7 cells.
