# Supplementary material for: Genomics of Signaling Crosstalk of Estrogen Receptor α in Breast Cancer Cells
Source: PLoS One. 2008 Mar 26;3(3):e1859. doi: 10.1371/journal.pone.0001859 (PMC2268000; doi:10.1371/journal.pone.0001859)
Supplement: Figure S2 — (0.11 MB PDF) [file pone.0001859.s003.pdf]

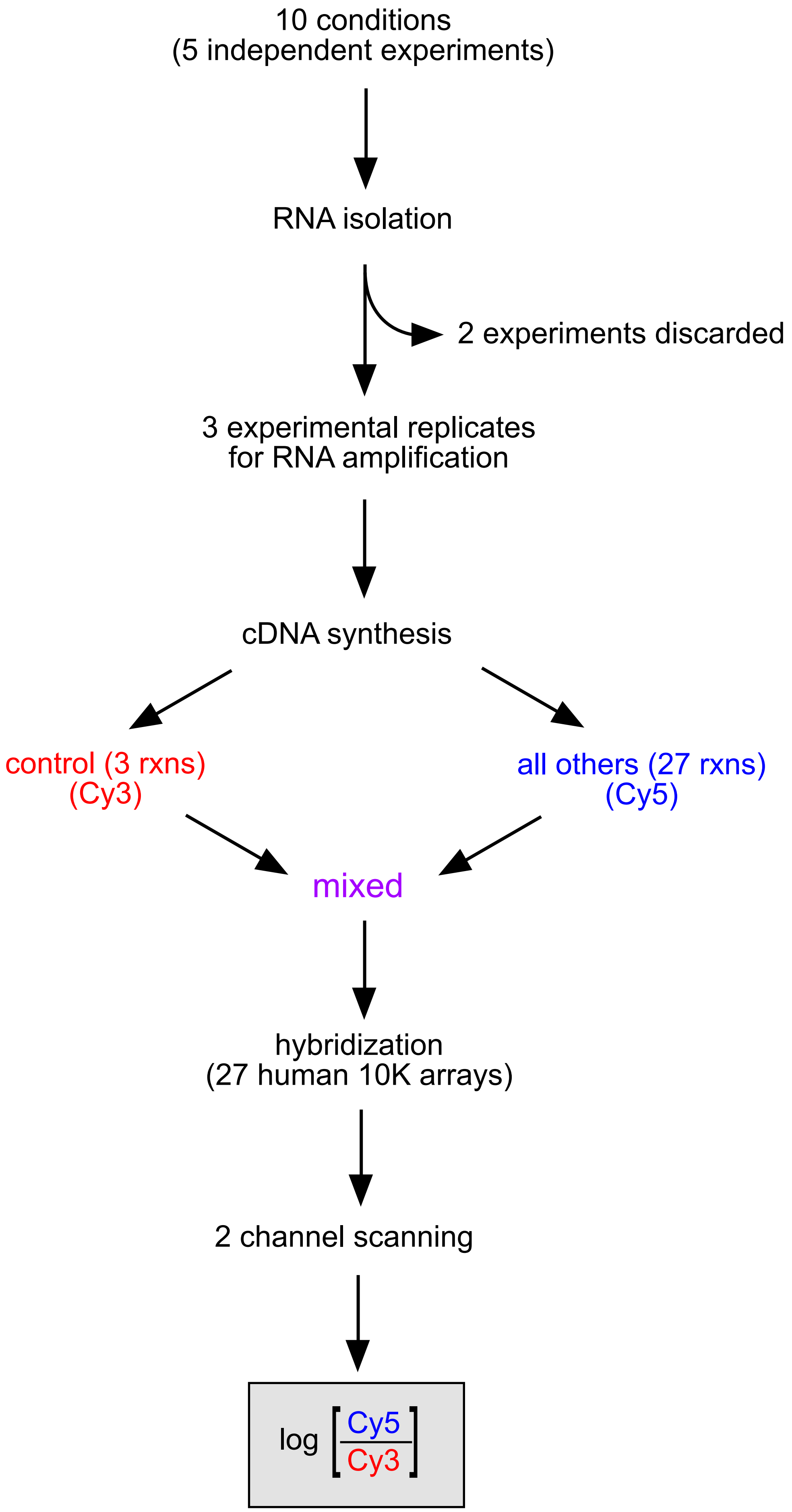

Supplementary Figure S2

Scheme of experimental flow from cell culture treatments to scanning of microarrays.
