# Supplementary material for: Genomics of Signaling Crosstalk of Estrogen Receptor α in Breast Cancer Cells
Source: PLoS One. 2008 Mar 26;3(3):e1859. doi: 10.1371/journal.pone.0001859 (PMC2268000; doi:10.1371/journal.pone.0001859)
Supplement: Table S1 — (0.03 MB PDF) [file pone.0001859.s004.pdf]

Table S1

Microarray treatment conditions

	E2	OHT	ICI	growth factors	cAMP
control					
2	+				
3		+			
4			+		
5				+	
6		+		+	
7			+	+	
8					+
9		+			+
10			+		+
