# Supplementary material for: Genomics of Signaling Crosstalk of Estrogen Receptor α in Breast Cancer Cells
Source: PLoS One. 2008 Mar 26;3(3):e1859. doi: 10.1371/journal.pone.0001859 (PMC2268000; doi:10.1371/journal.pone.0001859)
Supplement: Table S2 — (0.11 MB PDF) [file pone.0001859.s005.pdf]

Table S2

List of genes regulated by E2, cAMP or EGF/IGFI (≥ 2-fold)

GenBank	Symbol	Description	UniGene	Locus Link
**Estrogen-regulated target genes (90 of 220)**				
NM_005688	ABCC5	ATP-binding cassette, sub-family C (CFTR/MRP), member 5	Hs.22010	10057
NM_030767	AKNA	AT-hook transcription factor	Hs.159578	80709
NM_003568	ANXA9	Annexin A9	Hs.430324	8416
NM_001657	AREG	Amphiregulin (schwannoma-derived growth factor)	Hs.270833	374
NM_001661	ARF4L	ADP-ribosylation factor 4-like	Hs.183153	379
NM_000633 NM_000657	BCL2	B-cell CLL/lymphoma 2	Hs.79241	596
NM_031453	C10orf45	Chromosome 10 open reading frame 45	Hs.103378	83641
NM_001584	C11orf8	Chromosome 11 open reading frame 8	Hs.432000	744
NM_173481	C19orf21	Chromosome 19 open reading frame 21	Hs.439180	126353
NM_175931 NM_005187	CBFA2T3	Core-binding factor, runt domain, alpha subunit 2; translocated to, 3	Hs.110099	863
NM_004354	CCNG2	Cyclin G2	Hs.13291	901
NM_004143	CITED1	Cbp/p300-interacting transactivator, with Glu/Asp-rich carboxy-terminal domain, 1	Hs.40403	4435
NM_199168 NM_000609	CXCL12	Chemokine (C-X-C motif) ligand 12 (stromal cell-derived factor 1)	Hs.436042	6387
NM_000104	CYP1B1	Cytochrome P450, family 1, subfamily B, polypeptide 1	Hs.154654	1545
NM_004398	DDX10	[Other matche(s): Hs.41706] DEAD (Asp-Glu-Ala-Asp) box polypeptide 10	Hs.41706	1662
XM_291277	DKFZp761 P0423	Hypothetical protein DKFZp761P0423	Hs.276341	157285
NM_001952 NM_198325 NM_198258 NM_198257 NM_198256 NM_212540	E2F6	E2F transcription factor 6	Hs.135465	1876
NM_015516	E2IG4	Hypothetical protein, estradiol-induced	Hs.8361	25987
NM_182685 NM_004428	EFNA1	Ephrin-A1	Hs.399713	1942
NM_004430	EGR3	Early growth response 3	Hs.74088	1960
NM_005235	ERBB4	V-erb-a erythroblastic leukemia viral oncogene homolog 4 (avian)	Hs.7888	2066
	FARP1	FERM, RhoGEF (ARHGEF) and pleckstrin domain protein 1 (chondrocyte-derived)	Hs.403917	10160
NM_018000	FLJ10116	Hypothetical protein FLJ10116	Hs.79741	55686
NM_024871	FLJ12748	Hypothetical protein FLJ12748	Hs.203013	79929
NM_024563	FLJ14054	Hypothetical protein FLJ14054	Hs.13528	79614
NM_025069	FLJ14299	[Other matche(s): Hs.283869] Hypothetical protein FLJ14299	Hs.288042	80139
NM_019000	FLJ20152	Hypothetical protein FLJ20152	Hs.82273	54463
NM_017786	FLJ20366	Hypothetical protein FLJ20366	Hs.354793	55638
NM_198461	FLJ45273	FLJ45273 protein	Hs.30646	164832
NM_005252	FOS	V-fos FBJ murine osteosarcoma viral oncogene homolog	Hs.25647	2353
NM_001453	FOXC1	Forkhead box C1	Hs.348883	2296
NM_006350 NM_013409	FST	Follistatin	Hs.9914	10468
NM_012296 NM_080491	GAB2	GRB2-associated binding protein 2	Hs.30687	9846
NM_000816 NM_198904 NM_198903	GABRG2	Gamma-aminobutyric acid (GABA) A receptor, gamma 2	Hs.7195	2566

NM_014668<br>NM_148903<br>NM_033090	GREB1	GREB1 protein	Hs.438037	9687
NM_152451	Gup1	GRINL1A complex upstream protein	Hs.50841	145781
NM_002110	HCK	Hemopoietic cell kinase	Hs.443441	3055
NM_007069	HRASLS3	HRAS-like suppressor 3	Hs.528308	11145
NM_014365	HSPB8	Heat shock 22kDa protein 8	Hs.111676	26353
NM_001552	IGFBP4	Insulin-like growth factor binding protein 4	Hs.1516	3487
NM_000877	IL1R1	Interleukin 1 receptor, type I	Hs.82112	3554
NM_004973	JARID2	Jumonji, AT rich interactive domain 2	Hs.40154	3720
NM_004982	KCNJ8	Potassium inwardly-rectifying channel, subfamily J, member 8	Hs.102308	3764
NM_003740	KCNK5	Potassium channel, subfamily K, member 5	Hs.444448	8645
NM_020832	KIAA1441	KIAA1441 protein	Hs.186756	57592
NM_004235	KLF4	Kruppel-like factor 4 (gut)	Hs.376206	9314
NM_020163	LOC56920	Semaphorin sem2	Hs.59729	56920
NM_005375	MYB	V-myb myeloblastosis viral oncogene homolog (avian)	Hs.407830	4602
XM_034274	MYBL1	V-myb myeloblastosis viral oncogene homolog (avian)-like 1	Hs.300592	4603
NM_000909	NPY1R	Neuropeptide Y receptor Y1	Hs.169266	4886
NM_152745	NXPH1	Neurexophilin 1	Hs.487565	30010
NM_176871<br>NM_198042<br>NM_021630	PDLIM2	PDZ and LIM domain 2 (mystique)	Hs.521443	64236
NM_014476	PDLIM3	PDZ and LIM domain 3	Hs.71719	27295
NM_002614	PDZK1	PDZ domain containing 1	Hs.15456	5174
NM_000926	PGR	Progesterone receptor	Hs.368072	5241
NM_001002837<br>NM_014422	PIB5PA	Phosphatidylinositol (4,5) bisphosphate 5-phosphatase, A	Hs.21492	27124
NM_003557	PIP5K1A	[Other matche(s): Hs.537887] Phosphatidylinositol-4-phosphate 5-kinase, type I, alpha	Hs.149255	8394
NM_021127	PMAIP1	Phorbol-12-myristate-13-acetate-induced protein 1	Hs.96	5366
NM_005397	PODXL	Podocalyxin-like	Hs.16426	5420
NM_004878<br>NM_198797	PTGES	Prostaglandin E synthase	Hs.146688	9536
NM_016339	RAPGEFL1	Rap guanine nucleotide exchange factor (GEF)-like 1	Hs.158530	51195
NM_005739	RASGRP1	RAS guanyl releasing protein 1 (calcium and DAG-regulated)	Hs.189527	10125
NM_032918	RERG	RAS-like, estrogen-regulated, growth inhibitor	Hs.416854	85004
NM_020630<br>NM_020975	RET	Ret proto-oncogene (multiple endocrine neoplasia and medullary thyroid carcinoma 1, Hirschsprung disease)	Hs.350321	5979
NM_002928	RGS16	Regulator of G-protein signalling 16	Hs.413297	6004
NM_004155	SERPINB9	Serine (or cysteine) proteinase inhibitor, clade B (ovalbumin), member 9	Hs.104879	5272
NM_005627	SGK	Serum/glucocorticoid regulated kinase	Hs.296323	6446
NM_013257<br>NM_170709	SGKL	Serum/glucocorticoid regulated kinase-like	Hs.380877	23678
NM_003051	SLC16A1	Solute carrier family 16 (monocarboxylic acid transporters), member 1	Hs.75231	6566<br>246182
NM_003059	SLC22A4	Solute carrier family 22 (organic cation transporter), member 4	Hs.441130	6583
NM_003060	SLC22A5	Solute carrier family 22 (organic cation transporter), member 5	Hs.15813	6584
NM_003486	SLC7A5	Solute carrier family 7 (cationic amino acid transporter, y+ system), member 5	Hs.184601	8140
NM_004252	SLC9A3R1	Solute carrier family 9 (sodium/hydrogen exchanger), isoform 3 regulator 1	Hs.396783	9368
NM_005634	SOX3	SRY (sex determining region Y)-box 3	Hs.157429	6658
NM_021943	TEX27	Testis expressed sequence 27	Hs.6120	60685
NM_003222	TFAP2C	Transcription factor AP-2 gamma (activating enhancer binding protein 2 gamma)	Hs.440411	7022

NM_003236	TGFA	Transforming growth factor, alpha	Hs.170009	7039
NM_004613<br>NM_198951	TGM2	Transglutaminase 2 (C polypeptide, protein-glutamine-gamma-glutamyltransferase)	Hs.458353	7052
NM_005655	TIEG	TGFB inducible early growth response	Hs.82173	7071
NM_005077	TLE1	Transducin-like enhancer of split 1 (E(sp1) homolog, Drosophila)	Hs.406491	7088
NM_002546	TNFRSF11B	Tumor necrosis factor receptor superfamily, member 11b (osteoprotegerin)	Hs.81791	4982
NM_014452	TNFRSF21	Tumor necrosis factor receptor superfamily, member 21	Hs.159651	27242
NM_006670	TPBG	Trophoblast glycoprotein	Hs.82128	7162
NM_003287<br>NM_001003397<br>NM_001003396<br>NM_001003395	TPD52L1	Tumor protein D52-like 1	Hs.16611	7164
	UBE2D3	Ubiquitin-conjugating enzyme E2D 3 (UBC4/5 homolog, yeast)	Hs.472031	7323
NM_021147	UNG2	Uracil-DNA glycosylase 2	Hs.3041	10309
NM_015113	ZZEF1	Zinc finger, ZZ-type with EF hand domain 1	Hs.172179	23140
		Transcribed locus	Hs.10172	NA
		NBR2	Hs.463242	NA
		[Other matche(s): Hs.442160] Transcribed locus	Hs.442160	NA

cAMP-regulated target genes (22 of 220)

NM_182921<br>NM_182920<br>NM_020249	ADAMTS9	A disintegrin-like and metalloprotease (reprolysin type) with thrombospondin type 1 motif, 9	Hs.127811	56999
NM_031453	C10orf45	Chromosome 10 open reading frame 45	Hs.103378	83641
NM_000587	C7	Complement component 7	Hs.78065	730
NM_000735	CGA	Glycoprotein hormones, alpha polypeptide	Hs.119689	1081
NM_001873	CPE	Carboxypeptidase E	Hs.75360	1363
NM_001326	CSTF3	Hypothetical protein LOC283267	Hs.180034	1479<br>283267
NM_014750	DLG7	Discs, large homolog 7 (Drosophila)	Hs.77695	9787
NM_001422<br>NM_198381	ELF5	E74-like factor 5 (ets domain transcription factor)	Hs.11713	2001
NM_005239	ETS2	V-ets erythroblastosis virus E26 oncogene homolog 2 (avian)	Hs.292477	2114
XM_375837	FAM34A	Family with sequence similarity 34, member A	Hs.407774	9926
NM_002064	GLRX	Glutaredoxin (thioltransferase)	Hs.28988	2745
NM_000878	IL2RB	Interleukin 2 receptor, beta	Hs.75596	3560
NM_014657	KIAA0406	KIAA0406 gene product	Hs.410618	9675
NM_002747	MAPK4	Mitogen-activated protein kinase 4	Hs.433728	5596
NM_006241	PPP1R2	Protein phosphatase 1, regulatory (inhibitor) subunit 2	Hs.442946	5504
NM_015150	RAFTLIN	Raft-linking protein	Hs.436432	23180
NM_002923	RGS2	Regulator of G-protein signalling 2, 24kDa	Hs.78944	5997
NM_006924	SFRS1	Splicing factor, arginine/serine-rich 1 (splicing factor 2, alternate splicing factor)	Hs.68714	6426
NM_183422<br>NM_006022	TGFB1I4	Transforming growth factor beta 1 induced transcript 4	Hs.114360	8848
NM_006472	TXNIP	Thioredoxin interacting protein	Hs.179526	10628
		Transcribed locus	Hs.509468	NA
		Transcribed locus	Hs.536794	NA

Growth factor-regulated target genes (130 of 220)

NM_003786<br>NM_020037<br>NM_020038	ABCC3	ATP-binding cassette, sub-family C (CFTR/MRP), member 3	Hs.90786	8714
NM_001613	ACTA2	Actin, alpha 2, smooth muscle, aorta	Hs.208641	59
NM_001614	ACTG1	Actin, gamma 1	Hs.14376	71;60
NM_001615	ACTG2	Actin, gamma 2, smooth muscle, enteric	Hs.403989	72

NM_001102	ACTN1	Actinin, alpha 1	Hs.119000	87
NM_182921<br>NM_182920<br>NM_020249	ADAMTS9	A disintegrin-like and metalloprotease (reprolysin type) with thrombospondin type 1 motif, 9	Hs.127811	56999
NM_001124	ADM	Adrenomedullin	Hs.441047	133
NM_000676	ADORA2B	Adenosine A2b receptor	Hs.45743	136
NM_005139	ANXA3	Annexin A3	Hs.442733	306
NM_001657	AREG	Amphiregulin (schwannoma-derived growth factor)	Hs.270833	374
NM_005168	ARHE	Ras homolog gene family, member E	Hs.6838	390
NM_001677<br>NM_001001787	ATP1B1	ATPase, Na+/K+ transporting, beta 1 polypeptide	Hs.274479	481
NM_000332	ATXN1	Ataxin 1	Hs.434961	6310
NM_182962<br>NM_001165	BIRC3	Baculoviral IAP repeat-containing 3	Hs.127799	330
NM_004338<br>NM_181482<br>NM_181483<br>NM_181481<br>NM_001003675<br>NM_001003674	C18orf1	Chromosome 18 open reading frame 1	Hs.285091	753
NM_020130	C8orf4	Chromosome 8 open reading frame 4	Hs.283683	56892
NM_004342<br>NM_033138<br>NM_033139<br>NM_033140<br>NM_033157	CALD1	Caldesmon 1	Hs.443811	800
NM_001233<br>NM_198212	CAV2	Caveolin 2	Hs.139851	858
NM_005436	CCDC6	Coiled-coil domain containing 6	Hs.446360	8030
NM_003914	CCNA1	Cyclin A1	Hs.417050	8900
NM_004354	CCNG2	Cyclin G2	Hs.13291	901
NM_001790<br>NM_022809	CDC25C	Cell division cycle 25C	Hs.656	995
NM_006383	CIB2	Calcium and integrin binding family member 2	Hs.129867	10518
NM_021101	CLDN1	Claudin 1	Hs.7327	9076
NM_004078	CSRP1	Cysteine and glycine-rich protein 1	Hs.108080	1465
NM_001901	CTGF	Connective tissue growth factor	Hs.410037	1490
NM_001338	CXADR	Coxsackie virus and adenovirus receptor	Hs.79187	1525
NM_014326	DAPK2	Death-associated protein kinase 2	Hs.129208	23604
NM_001954<br>NM_013993<br>NM_013994	DDR1	[Other matche(s): Hs.154299] Discoidin domain receptor family, member 1	Hs.423573	780
NM_001925	DEFA4	Defensin, alpha 4, corticostatin	Hs.2582	1669
NM_003676<br>NM_144780	DEGS	Degenerative spermatocyte homolog, lipid desaturase (Drosophila)	Hs.299878	8560
NM_003676<br>NM_144780	DEGS	Degenerative spermatocyte homolog, lipid desaturase (Drosophila)	Hs.299878	8560
NM_012242	DKK1	Dickkopf homolog 1 (Xenopus laevis)	Hs.40499	22943
NM_001387	DPYSL3	Dihydropyrimidinase-like 3	Hs.150358	1809
NM_198057<br>NM_004089	DSIPI	Delta sleep inducing peptide, immunoreactor	Hs.420569	1831
NM_183380<br>NM_001723<br>NM_020388<br>NM_015548	DST	Dystonin	Hs.443518	667 7943
NM_001945	DTR	Diphtheria toxin receptor (heparin-binding epidermal growth factor-like growth factor)	Hs.799	1839
NM_001394<br>NM_057158	DUSP4	Dual specificity phosphatase 4	Hs.417962	1846
NM_022351	EFCBP1	EF hand calcium binding protein 1	Hs.302754	64168
NM_032571<br>NM_152939	EMR3	Egf-like module containing, mucin-like, hormone receptor-like 3	Hs.295626	84658
NM_006208	ENPP1	Ectonucleotide pyrophosphatase/phosphodiesterase 1	Hs.213840	5167

NM_001982	ERBB3	V-erb-b2 erythroblastic leukemia viral oncogene homolog 3 (avian)	Hs.306251	2065
NM_012291	ESPL1	Extra spindle poles like 1 (S. cerevisiae)	Hs.153479	9700
NM_005239	ETS2	V-ets erythroblastosis virus E26 oncogene homolog 2 (avian)	Hs.292477	2114
NM_005797 NM_144765	EVA1	Epithelial V-like antigen 1	Hs.116651	10205
NM_032270	FAD158	Factor for adipocyte differentiation 158	Hs.412836	84230
NM_001450 NM_201555 NM_201557 NM_201556	FHL2	Four and a half LIM domains 2	Hs.8302	2274
NM_018250	FLJ10871	Hypothetical protein FLJ10871	Hs.15562	55756
NM_024871	FLJ12748	Hypothetical protein FLJ12748	Hs.203013	79929
NM_017786	FLJ20366	Hypothetical protein FLJ20366	Hs.354793	55638
NM_198461	FLJ45273	FLJ45273 protein	Hs.30646	164832
NM_212482 NM_212478 NM_212476 NM_212475 NM_212474 NM_054034 NM_002026	FN1	Fibronectin 1	Hs.418138	2335
NM_005252	FOS	V-fos FBJ murine osteosarcoma viral oncogene homolog	Hs.25647	2353
NM_012296 NM_080491	GAB2	GRB2-associated binding protein 2	Hs.30687	9846
NM_000816 NM_198904 NM_198903	GABRG2	Gamma-aminobutyric acid (GABA) A receptor, gamma 2	Hs.7195	2566
NM_002053	GBP1	Guanylate binding protein 1, interferon-inducible, 67kDa	Hs.62661	2633
NM_004120	GBP2	Guanylate binding protein 2, interferon-inducible	Hs.386567	2634
NM_001490	GCNT1	Glucosaminyl (N-acetyl) transferase 1, core 2 (beta-1,6-N-acetylglucosaminyltransferase)	Hs.434857	2650
NM_000165	GJA1	Gap junction protein, alpha 1, 43kDa (connexin 43)	Hs.74471	2697
NM_177551	GPR109A	G protein-coupled receptor 109A	Hs.137555	338442
NM_012257	HBP1	HMG-box transcription factor 1	Hs.437606	26959
NM_000859	HMGCR	3-hydroxy-3-methylglutaryl-Coenzyme A reductase	Hs.11899	3156
NM_002130	HMGCS1	3-hydroxy-3-methylglutaryl-Coenzyme A synthase 1 (soluble)	Hs.397729	3157
XM_048898	HSPA12A	Heat shock 70kDa protein 12A	Hs.372597	259217
NM_014365	HSPB8	Heat shock 22kDa protein 8	Hs.111676	26353
NM_001550	IFRD1	Interferon-related developmental regulator 1	Hs.7879	3475
NM_000877	IL1R1	Interleukin 1 receptor, type I	Hs.82112	3554
NM_000418	IL4R	Interleukin 4 receptor	Hs.75545	3566
NM_002203	ITGA2	Integrin, alpha 2 (CD49B, alpha 2 subunit of VLA-2 receptor)	Hs.387725	3673
NM_002210	ITGAV	Integrin, alpha V (vitronectin receptor, alpha polypeptide, antigen CD51)	Hs.436873	3685
NM_015330	KIAA0376	KIAA0376 protein	Hs.4791	23384
NM_014772	KIAA0427	[Other matche(s): Hs.64096] KIAA0427	Hs.64096	9811
NM_005733	KIF20A	Kinesin family member 20A	Hs.153546	10112
NM_004235	KLF4	Kruppel-like factor 4 (gut)	Hs.376206	9314
NM_002309	LIF	Leukemia inhibitory factor (cholinergic differentiation factor)	Hs.2250	3976 91370
NM_005578	LPP	LIM domain containing preferred translocation partner in lipoma	Hs.180398	4026
NM_003954	MAP3K14	[Other matche(s): Hs.172199] Mitogen-activated protein kinase kinase kinase 14	Hs.440315	9020
NM_002381	MATN3	Matrilin 3	Hs.6985	4148
NM_144683	MGC23280	Hypothetical protein MGC23280	Hs.5163	147015

NM_000381<br>NM_033291<br>NM_033290	MID1	Midline 1 (Opitz/BBB syndrome)	Hs.27695	4281
NM_005938	MLLT7	Myeloid/lymphoid or mixed-lineage leukemia (trithorax homolog, Drosophila); translocated to, 7	Hs.239663	4303
NM_014751	MTSS1	Metastasis suppressor 1	Hs.77694	9788
NM_002473	MYH9	Myosin, heavy polypeptide 9, non-muscle	Hs.146550	4627
NM_004148	NINJ1	Ninjurin 1	Hs.11342	4814
NM_005010	NRCAM	Neuronal cell adhesion molecule	Hs.7912	4897
NM_005387<br>NM_016320<br>NM_139132<br>NM_139131	NUP98	Nucleoporin 98kDa	Hs.112255	4928
NM_002533<br>NM_206840	NVL	Nuclear VCP-like	Hs.58927	4931
NM_002611	PDK2	Pyruvate dehydrogenase kinase, isoenzyme 2	Hs.92261	5164
NM_003311	PHLDA2	Pleckstrin homology-like domain, family A, member 2	Hs.154036	7262
NM_001002837<br>NM_014422	PIB5PA	Phosphatidylinositol (4,5) bisphosphate 5-phosphatase, A	Hs.21492	27124
NM_006832	PLEKHC1	Pleckstrin homology domain containing, family C (with FERM domain) member 1	Hs.270411	10979
NM_005032	PLS3	Plastin 3 (T isoform)	Hs.430166	5358
NM_016147	PME-1	Protein phosphatase methylesterase-1	Hs.63304	51400
NM_000956	PTGER2	Prostaglandin E receptor 2 (subtype EP2), 53kDa	Hs.2090	5732
NM_006868	RAB31	[Other matche(s): Hs.251531] RAB31, member RAS oncogene family	Hs.223025	11031
NM_006834	RAB32	RAB32, member RAS oncogene family	Hs.493406	10981
NM_003979	RAI3	Retinoic acid induced 3	Hs.194691	9052
NM_003702<br>NM_170587	RGS20	Regulator of G-protein signalling 20	Hs.141492	8601
NM_005060<br>NM_001001523	RORC	RAR-related orphan receptor C	Hs.528667	6097
NM_004586	RPS6KA3	Ribosomal protein S6 kinase, 90kDa, polypeptide 3	Hs.188361	6197
NM_004186	SEMA3F	Sema domain, immunoglobulin domain (Ig), short basic domain, secreted, (semaphorin) 3F	Hs.32981	6405
NM_001046	SLC12A2	Solute carrier family 12 (sodium/potassium/chloride transporters), member 2	Hs.110736	6558
NM_003051	SLC16A1	Solute carrier family 16 (monocarboxylic acid transporters), member 1	Hs.75231	6566<br>246182
NM_005075<br>NM_021094<br>NM_134431	SLCO1A2	Solute carrier organic anion transporter family, member 1A2	Hs.46440	6579
NM_005902	SMAD3	SMAD, mothers against DPP homolog 3 (Drosophila)	Hs.288261	4088
NM_182965<br>NM_021972	SPHK1	Sphingosine kinase 1	Hs.68061	8877
NM_005086	SSPN	Sarcospan (Kras oncogene-associated gene)	Hs.183428	8082
NM_003186<br>NM_001001522	TAGLN	Transgelin	Hs.512705	6876
NM_015472	TAZ	Transcriptional co-activator with PDZ-binding motif	Hs.24341	25937
NM_003239	TGFB3	Transforming growth factor, beta 3	Hs.2025	7043
NM_002546	TNFRSF11B	Tumor necrosis factor receptor superfamily, member 11b (osteoprotegerin)	Hs.81791	4982
NM_016639	TNFRSF12A	Tumor necrosis factor receptor superfamily, member 12A	Hs.355899	51330
NM_014452	TNFRSF21	Tumor necrosis factor receptor superfamily, member 21	Hs.159651	27242
NM_003722	TP73L	Tumor protein p73-like	Hs.137569	8626
NM_003290	TPM4	Tropomyosin 4	Hs.419445	7171<br>7170
NM_003379	VIL2	Villin 2 (ezrin)	Hs.403997	7430

NM_021729	VPS11	[Other matche(s): Hs.274230] Vacuolar protein sorting 11 (yeast)	Hs.234282	55823
NM_031477	YPEL3	Yippee-like 3 (Drosophila)	Hs.271599	83719
NM_199450<br>NM_199452<br>NM_199451	ZNF365	Zinc finger protein 365	Hs.22653	22891<br>283045
NM_003461	ZYX	Zyxin	Hs.75873	7791
NM_015113	ZZEF1	Zinc finger, ZZ-type with EF hand domain 1	Hs.172179	23140
		Homo sapiens, clone IMAGE:5535936, mRNA	Hs.535933	NA
		Transcribed locus	Hs.467411	NA
		CDNA FLJ14942 fis, A-PLACE1011185	Hs.506182	NA
		[Other matche(s): Hs.270374]	Hs.270374	NA
		NBR2	Hs.463242	NA
		Full length insert cDNA clone ZD75H06	Hs.527013	NA
		Transcribed locus	Hs.538042	NA
		CDNA FLJ14942 fis, A-PLACE1011185	Hs.506182	NA
		Transcribed locus	Hs.467411	NA
