# Supplementary material for: Genomics of Signaling Crosstalk of Estrogen Receptor α in Breast Cancer Cells
Source: PLoS One. 2008 Mar 26;3(3):e1859. doi: 10.1371/journal.pone.0001859 (PMC2268000; doi:10.1371/journal.pone.0001859)
Supplement: Table S3 — (0.06 MB PDF) [file pone.0001859.s006.pdf]

Table S3

List of cAMP/GF-regulated genes affected by ICI (>20%)

Genbank	Symbol	Description	UniGene	Cluster
**GF-regulated target genes (65 of 623)**				
NM_001634	AMD1	Adenosylmethionine decarboxylase 1	Hs.159118	I
NM_016101	CGI-37	Comparative gene identification transcript 37	Hs.501513	I
NM_018000	FLJ10116	Hypothetical protein FLJ10116	Hs.79741	I
NM_014365	HSPB8	Heat shock 22kDa protein 8	Hs.111676	I
NM_181339<br>NM_006850	IL24	Interleukin 24	Hs.411311	I
NM_032435	KIAA1804	Mixed lineage kinase 4	Hs.50883	I
	LOC285958	Hypothetical protein LOC285958	Hs.25892	I
NM_021127	PMAIP1	Phorbol-12-myristate-13-acetate-induced protein 1	Hs.96	I
NM_021127	PMAIP1	Phorbol-12-myristate-13-acetate-induced protein 1	Hs.96	I
NM_002928	RGS16	Regulator of G-protein signalling 16	Hs.413297	I
NM_006924	SFRS1	Splicing factor, arginine/serine-rich 1 (splicing factor 2, alternate splicing factor)	Hs.68714	I
NM_006276	SFRS7	Splicing factor, arginine/serine-rich 7, 35kDa	Hs.309090	I
NM_004085	TIMM8A	Translocase of inner mitochondrial membrane 8 homolog A (yeast)	Hs.447877	I
NM_017786	FLJ20366	Hypothetical protein FLJ20366	Hs.354793	II
NM_003051	SLC16A1	Solute carrier family 16 (monocarboxylic acid transporters), member 1	Hs.75231	II
		NBR2	Hs.463242	II
NM_182921<br>NM_182920<br>NM_020249	ADAMTS9	A disintegrin-like and metalloprotease (reprolysin type) with thrombospondin type 1 motif, 9	Hs.127811	III
NM_001657	AREG	Amphiregulin (schwannoma-derived growth factor)	Hs.270833	III
NM_001657	AREG	Amphiregulin (schwannoma-derived growth factor)	Hs.270833	III
NM_024871	FLJ12748	Hypothetical protein FLJ12748	Hs.203013	III
NM_015113	ZZEF1	Zinc finger, ZZ-type with EF hand domain 1	Hs.172179	III
NM_001124	ADM	Adrenomedullin	Hs.441047	IV
NM_030978	ARPC5L	Actin related protein 2/3 complex, subunit 5-like	Hs.132499	IV
NM_182962<br>NM_001165	BIRC3	Baculoviral IAP repeat-containing 3	Hs.127799	IV
NM_001821	CHML	Choroideremia-like (Rab escort protein 2)	Hs.534313	IV
NM_001394<br>NM_057158	DUSP4	Dual specificity phosphatase 4	Hs.417962	IV
NM_022351	EFCBP1	EF hand calcium binding protein 1	Hs.302754	IV
NM_004094	EIF2S1	Eukaryotic translation initiation factor 2, subunit 1 alpha, 35kDa	Hs.151777	IV
XM_061871	FAT3	FAT tumor suppressor homolog 3 (Drosophila)	Hs.98523	IV
NM_198461	FLJ45273	FLJ45273 protein	Hs.30646	IV
NM_212482<br>NM_212478<br>NM_212476<br>NM_212475<br>NM_212474<br>NM_054034<br>NM_002026	FN1	Fibronectin 1	Hs.418138	IV
NM_015330	KIAA0376	KIAA0376 protein	Hs.4791	IV
NM_002297	LCN1	Lipocalin 1 (tear prealbumin)	Hs.2099	IV
NM_006575<br>NM_198794	MAP4K5	Mitogen-activated protein kinase kinase kinase kinase 5	Hs.246970	IV
NM_032717	MGC11324	Hypothetical protein MGC11324	Hs.99196	IV
NM_182981<br>NM_182980<br>NM_013370	OKL38	Pregnancy-induced growth inhibitor	Hs.528383	IV
NM_003722	TP73L	Tumor protein p73-like	Hs.137569	IV

NM_021729	VPS11	Vacuolar protein sorting 11 (yeast)	Hs.234282	IV
NM_014668<br>NM_148903<br>NM_033090	GREB1	GREB1 protein	Hs.438037	V
NM_002614	PDZK1	PDZ domain containing 1	Hs.15456	V
NM_003225	TFF1	Trefoil factor 1 (breast cancer, estrogen-inducible sequence expressed in)	Hs.350470	V
NM_004354	CCNG2	Cyclin G2	Hs.13291	VI
NM_001262<br>NM_078626	CDKN2C	Cyclin-dependent kinase inhibitor 2C (p18, inhibits CDK4)	Hs.4854	VI
NM_198057<br>NM_004089	DSIPI	Delta sleep inducing peptide, immunoreactor	Hs.420569	VI
NM_182685<br>NM_004428	EFNA1	Ephrin-A1	Hs.399713	VI
NM_001982	ERBB3	V-erb-b2 erythroblastic leukemia viral oncogene homolog 3 (avian)	Hs.306251	VI
NM_207336	EZI	Likely ortholog of mouse zinc finger protein EZI	Hs.112158	VI
NM_052943	FAM46B	Family with sequence similarity 46, member B	Hs.59771	VI
NM_152288	MGC13024	Hypothetical protein MGC13024	Hs.333488	VI
NM_005378	MYCN	V-myc myelocytomatosis viral related oncogene, neuroblastoma derived (avian)	Hs.25960	VI
NM_183422<br>NM_006022	TGFB1I4	Transforming growth factor beta 1 induced transcript 4	Hs.114360	VI
		Transcribed locus	Hs.517231	VI
		Transcribed locus	Hs.536054	VI
NM_152267	FLJ38628	Hypothetical protein FLJ38628	Hs.433156	VII
NM_002087	GRN	Granulin	Hs.180577	VII
NM_002824	PTMS	Parathymosin	Hs.446525	VII
NM_003944	SELENBP1	Selenium binding protein 1	Hs.334841	VII
NM_003331	TYK2	Tyrosine kinase 2	Hs.75516	VII
NM_014667	VGLL4	Vestigial like 4 (Drosophila)	Hs.155584	VII
NM_015481	ZNF385	Zinc finger protein 385	Hs.278422	VII
		Transcribed locus	Hs.480161	VII
NM_004433	ELF3	E74-like factor 3 (ets domain transcription factor, epithelial-specific)	Hs.67928	VIII
NM_001003684<br>NM_013387	HSPC051	Ubiquinol-cytochrome c reductase complex (7.2 kD)	Hs.284292	VIII
NM_020775	KIAA1324	Maba1	Hs.104696	VIII
NM_002885	RAP1GA1	RAP1, GTPase activating protein 1	Hs.433797	VIII

cAMP-regulated target genes (28 of 406)

NM_014583	LMCD1	LIM and cysteine-rich domains 1	Hs.279943	*
NM_015150	RAFTLIN	Raft-linking protein	Hs.436432	**
NM_014447	ARFIP1	ADP-ribosylation factor interacting protein 1 (arfaptin 1)	Hs.416089	I
NM_001832	CLPS	Colipase, pancreatic	Hs.1340	I
NM_003038	SLC1A4	Solute carrier family 1 (glutamate/neutral amino acid transporter), member 4	Hs.153307	I
NM_014467	SRPX2	Sushi-repeat-containing protein, X-linked 2	Hs.306339	I
		Transcribed locus	Hs.121070	I
		Transcribed locus	Hs.541338	I
NM_001936<br>NM_130797	DPP6	Dipeptidylpeptidase 6	Hs.390175	II
NM_181894<br>NM_000828<br>NM_007325	GRIA3	Glutamate receptor, ionotrophic, AMPA 3	Hs.377070	II
NM_002257	KLK1	Kallikrein 1, renal/pancreas/salivary	Hs.123107	II
NM_004546	NDUFB2	NADH dehydrogenase (ubiquinone) 1 beta subcomplex, 2, 8kDa	Hs.27262	II
NM_176894<br>NM_023914	P2RY13	Purinergic receptor P2Y, G-protein coupled, 13	Hs.386296	II
NM_000536	RAG2	Recombination activating gene 2	Hs.159376	II

NM_199415<br>NM_014948<br>NM_021826	UBCE7IP5	Likely ortholog of mouse ubiquitin conjugating enzyme 7 interacting protein 5	Hs.442605	II
NM_001661	ARF4L	ADP-ribosylation factor 4-like	Hs.183153	III
NM_000633<br>NM_000657	BCL2	B-cell CLL/lymphoma 2	Hs.79241	III
NM_000587	C7	Complement component 7	Hs.78065	III
NM_004443	EPHB3	EphB3	Hs.2913	III
NM_181353<br>NM_002165	ID1	Inhibitor of DNA binding 1, dominant negative helix-loop-helix protein	Hs.410900	III
NM_014657	KIAA0406	KIAA0406 gene product	Hs.410618	III
XM_044632	KIAA0556	KIAA0556 protein	Hs.30512	III
NM_000248<br>NM_198178<br>NM_198177<br>NM_006722<br>NM_198159<br>NM_198158	MITF	Microphthalmia-associated transcription factor	Hs.166017	III
NM_000112	SLC26A2	Solute carrier family 26 (sulfate transporter), member 2	Hs.302738	III
NM_021943	TEX27	Testis expressed sequence 27	Hs.6120	III
NM_003225	TFF1	Trefoil factor 1 (breast cancer, estrogen-inducible sequence expressed in)	Hs.350470	III
		CDNA FLJ34428 fis, clone HLUNG2000761	Hs.473191	III
		Transcribed locus	Hs.434969	III
