# Supplementary material for: Genomics of Signaling Crosstalk of Estrogen Receptor α in Breast Cancer Cells
Source: PLoS One. 2008 Mar 26;3(3):e1859. doi: 10.1371/journal.pone.0001859 (PMC2268000; doi:10.1371/journal.pone.0001859)
Supplement: Table S4 — (0.08 MB PDF) [file pone.0001859.s007.pdf]

Table S4

List of genes regulated by cAMP or EGF/IGFI for which OHT behaves as agonist (≥ 1.3-fold)

GenBank	Symbol	Description	UniGene	Locus Link
**cAMP-regulated genes for which OHT behaves as agonist (80 of 108)**				
NM_003488<br>NM_139275	AKAP1	A kinase (PRKA) anchor protein 1	Hs.78921	8165
NM_005163	AKT1	V-akt murine thymoma viral oncogene homolog 1	Hs.368861	207
NM_005876	APEG1	Aortic preferentially expressed protein 1	Hs.21639	10290
NM_014447	ARFIP1	ADP-ribosylation factor interacting protein 1 (arfaptin 1)	Hs.416089	27236
NM_005738<br>NM_212460	ARL4A	ADP-ribosylation factor-like 4A	Hs.245540	10124
NM_024626	B7-H4	Immune costimulatory protein B7-H4	Hs.36563	79679
NM_000465	BARD1	BRCA1 associated RING domain 1	Hs.54089	580
NM_182962<br>NM_001165	BIRC3	Baculoviral IAP repeat-containing 3	Hs.127799	330
NM_006820	C1orf29	Chromosome 1 open reading frame 29	Hs.389724	10964
NM_000715	C4BPA	Complement component 4 binding protein, alpha	Hs.1012	722
NM_005795	CALCRL	Calcitonin receptor-like	Hs.152175	10203
NM_001227<br>NM_033340<br>NM_033339<br>NM_033338	CASP7	Caspase 7, apoptosis-related cysteine protease	Hs.9216	840
NM_001797<br>NM_033664	CDH11	Cadherin 11, type 2, OB-cadherin (osteoblast)	Hs.443435	1009
NM_004143	CITED1	Cbp/p300-interacting transactivator, with Glu/Asp-rich carboxy-terminal domain, 1	Hs.40403	4435
NM_001832	CLPS	Colipase, pancreatic	Hs.1340	1208
NM_021151	CROT	Carnitine O-octanoyltransferase	Hs.125039	54677
NM_199168<br>NM_000609	CXCL12	Chemokine (C-X-C motif) ligand 12 (stromal cell-derived factor 1)	Hs.436042	6387
NM_199168<br>NM_000609	CXCL12	Chemokine (C-X-C motif) ligand 12 (stromal cell-derived factor 1)	Hs.436042	6387
NM_000104	CYP1B1	Cytochrome P450, family 1, subfamily B, polypeptide 1	Hs.154654	1545
NM_004397	DDX6	DEAD (Asp-Glu-Ala-Asp) box polypeptide 6	Hs.271541	1656<br>29007
NM_001936<br>NM_130797	DPP6	Dipeptidylpeptidase 6	Hs.390175	1804
NM_005235	ERBB4	V-erb-a erythroblastic leukemia viral oncogene homolog 4 (avian)	Hs.7888	2066
NM_005239	ETS2	V-ets erythroblastosis virus E26 oncogene homolog 2 (avian)	Hs.292477	2114
XM_376328	FAM13A1	Family with sequence similarity 13, member A1	Hs.442818	10144
	FARP1	FERM, RhoGEF (ARHGEF) and pleckstrin domain protein 1 (chondrocyte-derived)	Hs.403917	10160
NM_005245	FAT	FAT tumor suppressor homolog 1 (Drosophila)	Hs.166994	2195
NM_004469	FIGF	C-fos induced growth factor (vascular endothelial growth factor D)	Hs.11392	2277
NM_002019	FLT1	Fms-related tyrosine kinase 1 (vascular endothelial growth factor/vascular permeability factor receptor)	Hs.128271	2321
NM_006350<br>NM_013409	FST	Follistatin	Hs.9914	10468
NM_002064	GLRX	Glutaredoxin (thioltransferase)	Hs.28988	2745
NM_014668<br>NM_148903<br>NM_033090	GREB1	GREB1 protein	Hs.438037	9687

NM_181894<br>NM_000828<br>NM_007325	GRIA3	Glutamate receptor, ionotrophic, AMPA 3	Hs.377070	2892
NM_002140<br>NM_031263<br>NM_031262	HNRPK	Heterogeneous nuclear ribonucleoprotein K	Hs.307544	3190
NM_007069	HRASLS3	HRAS-like suppressor 3	Hs.528308	11145
NM_001003684<br>NM_013387	HSPC051	Ubiquinol-cytochrome c reductase complex (7.2 kD)	Hs.284292	29796
NM_001001887	IFIT1	Interferon-induced protein with tetratricopeptide repeats 1	Hs.20315	3434
NM_002201	ISG20	Interferon stimulated gene 20kDa	Hs.105434	3669
NM_006084	ISGF3G	Interferon-stimulated transcription factor 3, gamma 48kDa	Hs.1706	10379
NM_014657	KIAA0406	KIAA0406 gene product	Hs.410618	9675
XM_044632	KIAA0556	KIAA0556 protein	Hs.30512	23247
NM_015210	KIAA0802	KIAA0802	Hs.127716	23255<br>284219
NM_002257	KLK1	Kallikrein 1, renal/pancreas/salivary	Hs.123107	3816
NM_015315	LARP	Likely ortholog of mouse la related protein	Hs.6214	23367
NM_002297	LCN1	Lipocalin 1 (tear prealbumin)	Hs.2099	3933
NM_005584	MAB21L1	Mab-21-like 1 (C. elegans)	Hs.507743	4081
NM_005935	MLLT2	Myeloid/lymphoid or mixed-lineage leukemia (trithorax homolog, Drosophila); translocated to, 2	Hs.114765	4299
NM_032476	MRPS6	Mitochondrial ribosomal protein S6	Hs.268016	64968
NM_002462	MX1	Myxovirus (influenza virus) resistance 1, interferon-inducible protein p78 (mouse)	Hs.436836	4599
NM_005375	MYB	V-myb myeloblastosis viral oncogene homolog (avian)	Hs.407830	4602
NM_004546	NDUFB2	NADH dehydrogenase (ubiquinone) 1 beta subcomplex, 2, 8kDa	Hs.27262	4708
NM_005599	NHLH2	Nescient helix loop helix 2	Hs.46296	4808
NM_018376	NIPSNAP3B	Nipsnap homolog 3B (C. elegans)	Hs.202393	55335<br>286367
NM_173158<br>NM_173157<br>NM_002135	NR4A1	Nuclear receptor subfamily 4, group A, member 1	Hs.1119	3164
XM_375762	NTNG1	Netrin G1	Hs.298393	22854
NM_006703	NUDT3	Nudix (nucleoside diphosphate linked moiety X)-type motif 3	Hs.4815	11165
NM_016816<br>NM_002534	OAS1	2',5'-oligoadenylate synthetase 1, 40/46kDa	Hs.442936	4938
NM_176894<br>NM_023914	P2RY13	Purinergic receptor P2Y, G-protein coupled, 13	Hs.386296	53829
NM_176871<br>NM_198042<br>NM_021630	PDLIM2	PDZ and LIM domain 2 (mystique)	Hs.521443	64236
NM_181504<br>NM_181524<br>NM_181523	PIK3R1	Phosphoinositide-3-kinase, regulatory subunit, polypeptide 1 (p85 alpha)	Hs.6241	5295
NM_005398	PPP1R3C	Protein phosphatase 1, regulatory (inhibitor) subunit 3C	Hs.303090	5507
NM_006246	PPP2R5E	Protein phosphatase 2, regulatory subunit B (B56), epsilon isoform	Hs.173328	5529
NM_015150	RAFTLIN	Raft-linking protein	Hs.436432	23180
NM_000536	RAG2	Recombination activating gene 2	Hs.159376	5897
NM_002885	RAP1GA1	RAP1, GTPase activating protein 1	Hs.433797	5909
NM_019034	RHOF	Ras homolog gene family, member F (in filopodia)	Hs.512618	54509
NM_019845	RPRM	Reprimo, TP53 dependant G2 arrest mediator candidate	Hs.100890	56475
NM_005063	SCD	Stearoyl-CoA desaturase (delta-9-desaturase)	Hs.119597	6319
NM_014445	SERP1	Stress-associated endoplasmic reticulum protein 1	Hs.439874	27230
NM_003955	SOCS3	Suppressor of cytokine signaling 3	Hs.436943	9021
NM_014467	SRPX2	Sushi-repeat-containing protein, X-linked 2	Hs.306339	27286

NM_004613<br>NM_198951	TGM2	Transglutaminase 2 (C polypeptide, protein-glutamine-gamma-glutamyltransferase)	Hs.458353	7052
NM_001064	TKT	Transketolase (Wernicke-Korsakoff syndrome)	Hs.89643	7086
NM_006472	TXNIP	Thioredoxin interacting protein	Hs.179526	10628
NM_199415<br>NM_014948<br>NM_021826	UBCE7IP5	Likely ortholog of mouse ubiquitin conjugating enzyme 7 interacting protein 5	Hs.442605	22888<br>60493
		Transcribed locus	Hs.35125	NA
		Transcribed locus	Hs.541338	NA

EGF/IGFI-regulated genes for which OHT behaves as agonist (28 of 108)

NM_032501	ACAS2L	Acetyl-Coenzyme A synthetase 2 (AMP forming)-like	Hs.7218	84532
NM_001124	ADM	Adrenomedullin	Hs.441047	133
NM_001202<br>NM_130851<br>NM_130850	BMP4	Bone morphogenetic protein 4	Hs.68879	652
NM_020130	C8orf4	Chromosome 8 open reading frame 4	Hs.283683	56892
NM_006366	CAP2	CAP, adenylate cyclase-associated protein, 2 (yeast)	Hs.296341	10486
NM_021151	CROT	Carnitine O-octanoyltransferase	Hs.125039	54677
NM_000782	CYP24A1	Cytochrome P450, family 24, subfamily A, polypeptide 1	Hs.89663	1591
NM_001554	CYR61	Cysteine-rich, angiogenic inducer, 61	Hs.8867	3491
NM_021145	DMTF1	Cyclin D binding myb-like transcription factor 1	Hs.174941	9988
NM_005228<br>NM_201284<br>NM_201283<br>NM_201282	EGFR	Epidermal growth factor receptor (erythroblastic leukemia viral (v-erb-b) oncogene homolog, avian)	Hs.77432	1956
NM_004454	ETV5	Ets variant gene 5 (ets-related molecule)	Hs.43697	2119
NM_005245	FAT	FAT tumor suppressor homolog 1 (Drosophila)	Hs.166994	2195
NM_004496	FOXA1	Forkhead box A1	Hs.163484	3169
NM_004120	GBP2	Guanylate binding protein 2, interferon-inducible	Hs.386567	2634
NM_000841	GRM4	Glutamate receptor, metabotropic 4	Hs.429018	2914
NM_004262	HAT	Airway trypsin-like protease	Hs.132195	9407
NM_000188<br>NM_033496<br>NM_033497<br>NM_033498<br>NM_033500	HK1	Hexokinase 1	Hs.118625	3098
NM_001003684<br>NM_013387	HSPC051	Ubiquinol-cytochrome c reductase complex (7.2 kD)	Hs.284292	29796
NM_002201	ISG20	Interferon stimulated gene 20kDa	Hs.105434	3669
NM_007196<br>NM_144507<br>NM_144506<br>NM_144505	KLK8	Kallikrein 8 (neuropsin/ovasin)	Hs.104570	11202
NM_005935	MLLT2	Myeloid/lymphoid or mixed-lineage leukemia (trithorax homolog, Drosophila); translocated to, 2	Hs.114765	4299
XM_375762	NTNG1	Netrin G1	Hs.298393	22854
NM_002885	RAP1GA1	RAP1, GTPase activating protein 1	Hs.433797	5909
NM_004787	SLIT2	Slit homolog 2 (Drosophila)	Hs.29802	9353
NM_005900<br>NM_001003688	SMAD1	SMAD, mothers against DPP homolog 1 (Drosophila)	Hs.357304	4086
NM_005723	TM4SF9	Transmembrane 4 superfamily member 9	Hs.8037	10098
NM_003722	TP73L	Tumor protein p73-like	Hs.137569	8626
NM_001074	UGT2B7	UDP glycosyltransferase 2 family, polypeptide B7	Hs.10319	7364
