# Supplementary material for: Genomics of Signaling Crosstalk of Estrogen Receptor α in Breast Cancer Cells
Source: PLoS One. 2008 Mar 26;3(3):e1859. doi: 10.1371/journal.pone.0001859 (PMC2268000; doi:10.1371/journal.pone.0001859)
Supplement: Table S5 — (0.06 MB PDF) [file pone.0001859.s008.pdf]

Table S5-A

Summary of scan for common modules

	1 element	2 elements	3 elements
cAMP-induced	74	34	8
cAMP-repressed	120	67	3

Table S5-B

Statistics of genome-wide search for modules

	Number of Matches			Number of Promoters
	Total	(+) strand	(-) strand	
MI1	35	22	13	35
MI2	118	67	51	115
MI3	683	343	340	597
MR1	1339	661	678	1256

**Table**	**S5-C**			
**Significant GO groups found**				
	GO group	Number of genes (observed)	Number of genes (expected)	Gene Names
MI1	response to virus	2	0.06	*IFNA4, TLR8*
	cytokine receptor binding	2	0.08	*IFNA21, IFNA4*
	nuclease activity	2	0.2	*ERCC1, 3'HEXO*
MI2	small GTPase mediated signal transduction	8	1.3	*PLD2, RGS19, ARHGAP4, RAB15, RAPGEF3, RAB3B, RABL2B, RABL2A*
	GTPase activity	5	0.93	*EIF2S3, RAB3B, TUBB, RABL2B, RABL2A*
MI3	parturition	3	0.17	*PLA2G4B, CRHR1, OXT*
	G2 phase of mitotic cell cycle	2	0.1	*KPNA2, CHES1*
	L-serine metabolism	3	0.34	*SRR, CTBP2, CTBP1*
	epigenetic regulation of gene expression	5	1.01	*HDAC5, BRUNOL4, DNMT3A, DNMT3B, ARID1A*
	adenylate cyclase inhibiting pathway	6	1.37	*GABBR1, CRHR1, GNAS, GRM7, ADRB1, DRD2*
MR1	regulation of epidermal growth factor receptor activity	3	0.18	*NCK2, SNX6, SHC1*
	calcium-dependent cell-cell adhesion	3	0.24	*NLGN1, PCDHB5, PCDHB4*
	neuronal cell adhesion	2	0.18	*CTNND2, NCAM2*
	sphingomyelin biosynthesis	2	0.06	*TMEM23, MGC26963*
