# Supplementary material for: Genomics of Signaling Crosstalk of Estrogen Receptor α in Breast Cancer Cells
Source: PLoS One. 2008 Mar 26;3(3):e1859. doi: 10.1371/journal.pone.0001859 (PMC2268000; doi:10.1371/journal.pone.0001859)
Supplement: Table S6 — (0.03 MB PDF) [file pone.0001859.s009.pdf]

Table S6

Gene	Forward (5′-3′)	Reverse (5′-3′)
DNM3	GTTATCACCAAACTGGACCT	CTATGTCCTTCTGGCTTCTG
BCL2	GATGACTGAGTACCTGAACC	CCAGGAGAAATCAAACAGAG
TFF1	ATGGAGAACAAGGTGATCTG	ACCACAATTCTGTCTTTCAC
RGS16	AGTTCAAGAAGATCCGATCAG	TGGGTCTCATGGTCAATGTT
RAP1	GATCGGTTCTATCCTGTGCT	GCTTCTGATAAATGACGCCA
EPHB3	CTGCCACAATAACTTCTACC	GAGGTTTCATTCACATTGGA
CCNG2	TATTAGCCTTGTGCCTTCTC	CCAGTAGAAGAACTCAGTGTC
